# Phosphorylation of PPARγ Affects the Collective Motions of the PPARγ-RXRα-DNA Complex

**DOI:** 10.1371/journal.pone.0123984

**Published:** 2015-05-08

**Authors:** Justin A. Lemkul, Stephanie N. Lewis, Josep Bassaganya-Riera, David R. Bevan

**Affiliations:** 1 Department of Biochemistry, Virginia Tech, Blacksburg, Virginia, United States of America; 2 Nutritional Immunology & Molecular Medicine Laboratory, Virginia Bioinformatics Institute, Virginia Tech, Blacksburg, Virginia, United States of America; University of South Florida College of Medicine, UNITED STATES

## Abstract

Peroxisome-proliferator activated receptor-γ (PPARγ) is a nuclear hormone receptor that forms a heterodimeric complex with retinoid X receptor-α (RXRα) to regulate transcription of genes involved in fatty acid storage and glucose metabolism. PPARγ is a target for pharmaceutical intervention in type 2 diabetes, and insight into interactions between PPARγ, RXRα, and DNA is of interest in understanding the function and regulation of this complex. Phosphorylation of PPARγ by cyclin-dependent kinase 5 (Cdk5) has been shown to dysregulate the expression of metabolic regulation genes, an effect that is counteracted by PPARγ ligands. We applied molecular dynamics (MD) simulations to study the relationship between the ligand-binding domains of PPARγ and RXRα with their respective DNA-binding domains. Our results reveal that phosphorylation alters collective motions within the PPARγ-RXRα complex that affect the LBD-LBD dimerization interface and the AF-2 coactivator binding region of PPARγ.

## Introduction

PPARγ is a transcription factor within the nuclear hormone receptor family that forms a heterodimeric complex with RXRα to bind coactivator proteins that recruit additional transcription factors to PPRE sequences, which are generally located in enhancer regions far upstream from the target genes [1,2]. PPARγ has a structure that is typical of nuclear hormone receptors, containing an N-terminal A/B domain of unknown structure followed by two principally α-helical domains, a 12-helix ligand-binding domain (LBD) and a zinc-finger DNA-binding domain (DBD). There are two isoforms of PPARγ (PPARγ1 and PPARγ2), with PPARγ2 containing an N-terminal extension of 28 amino acids. The two isoforms are otherwise identical throughout the LBD and DBD. The N-terminal A/B domain contains a weakly conserved transcriptional activation region known as activation function-1 (AF-1) [3]. The LBD of PPARγ serves not only to bind endogenous ligands, but also to dimerize with RXRα and bind coactivator proteins in the AF-2 region. A recent structural study by Chandra et al. on PPARγ1 showed that the PPARγ LBD is intimately linked with both the RXRα LBD and DBD [4].

PPARγ is the “master regulator” of adipocyte differentiation and functions in many cellular pathways, including regulating lipid storage, cell proliferation, and inflammatory processes that are involved in immunity [5]. PPARγ is also a target for marketed antidiabetic drugs, as agonists binding to the LBD increase insulin sensitization [1,6]. A classic example of a drug that acts as a full agonist of PPARγ is rosiglitazone, a member of the thiazolidinedione (TZD) class of antidiabetic drugs. In 2010, the FDA placed restrictions on prescribing and dispensing rosiglitazone-based drugs due to concerns about increased cardiovascular disease in patients taking the drugs. Further analysis of the data led the FDA to remove those restrictions in 2014. The uncertainty of long-term effects on patient health suggests a continuing need for additional, novel drugs that target PPARγ, and a rational and informed approach to identifying drugs requires an understanding of molecular mechanisms of receptor activation.

At the molecular level, full agonists tend to bind to the PPARγ LBD in a polar region of the ligand-binding site, stabilizing helix H12 and the AF-2 region [7,8]. In contrast, partial agonists bind in the binding site entrance channel and an alternate site that stabilizes H3 [9,10]. These differences in binding orientation have stimulated a search for partial agonists with therapeutic potential, the rationale being that the somewhat weaker agonistic activity may also lead to fewer undesirable side effects [7,11] and that many synthetic compounds believed to be full agonists are truly partial agonists, in that they can elicit differences in gene expression patterns to address specific disease conditions [12]. Similarly, concerns that ligands designed to target PPARγ could also agonize or antagonize the PPARα and PPARβ/δ subtypes has led to efforts to identify subtype-selective agonists. A distinctly different approach to developing therapeutics that target PPARγ is to modulate post-translational modification, as discussed below.

Transcriptional activity of nuclear receptors can be regulated by post-translational modifications such as phosphorylation [13]. Phosphorylation of Ser112 in PPARγ2 in the N-terminal A/B domain inhibits ligand binding [14], despite the large spatial separation between Ser112 and the LBD. This finding implicates long-range collective dynamics and interdomain interactions in the function and regulation of PPARγ transcriptional activity. More recently, Choi et al. demonstrated that PPARγ2 can be phosphorylated by Cdk5 on Ser273 (Ser245 in the PPARγ1) in the LBD [15], dysregulating the expression of metabolic regulation genes, including adipsin and adiponectin [15]. However, not all PPARγ-regulated genes are affected, and PPARγ chromatin occupancy was unaffected, indicating that the effects of phosphorylation of Ser273 may result from altered coactivator binding rather than impeding DNA binding. Choi et al. further demonstrated that PPARγ ligands were capable of inhibiting phosphorylation [15], but that agonism was not a prerequisite for this effect [16]. Based on the structural work of Chandra et al. [4], it is clear that Ser273/245 makes close contact with the RXRα DBD, thus implicating protein-protein interactions and resulting dynamics as a target for regulation by phosphorylation and ultimately the ability of coactivator proteins to bind to the AF-2 region.

For this model to be correct, phosphorylated PPARγ (p-PPARγ) must accommodate binding to RXRα, therefore RXRα must be bound by retinoic acid to disassemble from its tetrameric storage form [17] to be available to interact with p-PPARγ. PPARγ agonists (full and partial) can restore some level of transcriptional activity in genes that are otherwise dysregulated by phosphorylation, implying that PPARγ ligands can bind to p-PPARγ and potentially counteract the effect of phosphorylation [15]. From the standpoint of targeting PPARγ with anti-diabetic compounds, it is also important to consider that PPARγ will exist in a mixture of states *in vivo*, both phosphorylated and non-phosphorylated, thus the dynamics of p-PPARγ bound to putative drug molecules are relevant to drug design.

In the present study, we applied molecular dynamics (MD) simulations to examine interactions among the components of the PPARγ1-RXRα-DNA complex. We undertook the present work for several reasons: to understand (i) how phosphorylation affects functional dynamics of the RXRα-PPARγ1 complex, (ii) the means by which partial agonists stabilize the complex, and (iii) how the interplay between ligand binding and phosphorylation impacts the dynamics of the RXRα-PPARγ complex. We hypothesized that phosphorylation within the LBD would alter the conformational ensemble of PPARγ, and that the dynamics would be further modulated by coactivators and bound ligands. Ultimately, these macromolecular interactions are likely to have implications for coactivator recruitment and interactions, thus contributing to information about expression of PPARγ-modulated genes and the larger role of allostery in the activity of nuclear hormone receptors. The present study focuses on the ternary PPARγ1-RXRα-DNA complex, using a crystallographic model solved by Chandra et al. [4]. For this reason, all residue numbers in this paper are given as they appear in PPARγ1, with Ser273 in PPARγ2 being equivalent to Ser245 here. By utilizing long MD simulations, we sought to provide the most complete picture to date of functional PPARγ dynamics with atomistic resolution.

## Results

**Fig 1:**
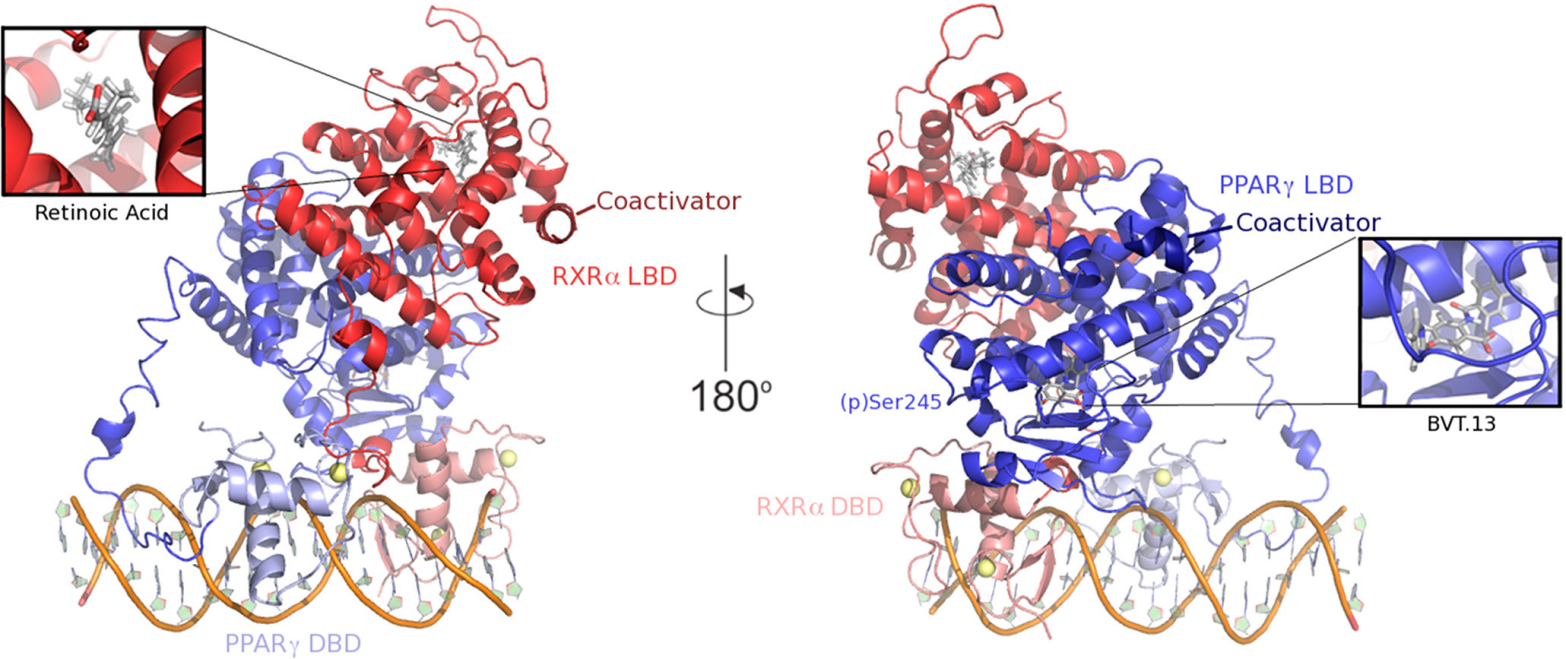
Components of the PPARγ-RXRα-DNA ternary complex. Protein structural domains (DBD, DNA-binding domain and LBD, ligand-binding domain) are indicated. Yellow spheres in the two DBD are Zn^2+^ ions. DNA is shown as a cartoon.

Given the model described above, we based our simulations on the crystal structure of the PPARγ-RXRα-DNA complex solved by Chandra et al. [4]. All complexes in our simulations contained PPARγ, RXRα, and DNA, with differences among the complexes consisting of phosphorylation state of Ser245 and presence or absence of ligands or coactivator peptides (see Methods, Table 1 and Fig 1). These different complexes allowed for an extensive analysis of the collective motions of the PPARγ-RXRα complex, especially those at the protein-protein interfaces, to assess any alterations to the dynamics of the complex, thus affecting interactions of PPARγ with DNA and coactivator peptides. Including the partial agonist (BVT.13) in the complex in some simulations was done in an effort to understand the mechanism underlying recovery of transcriptional activity in p-PPARγ that was observed by Choi et al. [15].

**Table 1:** Summary of the contents of each complex.

Complex	pSer245	Retinoic acid	BVT.13	Coactivator peptides
Apo				
Unbound				X
Holo		X	X	X
Phospho	X	X	X	X
Phospho-Unbound	X	X		X

All complexes contained PPARγ, RXRα, and DNA, with “X” indicating the presence of other components.

### Collective Motions Within the PPARγ-RXRα Complex

To analyze the low frequency motions of the PPARγ-RXRα complex, we performed principal components analysis (PCA) as described in the Methods. In all of the complexes, the most prominent collective motions within PPARγ involved the H2’-H3 loop and the hinge that connects the DBD and LBD. Subtle differences in the directions of these motions had implications for the dynamics of the AF-2 region of PPARγ and the dimerization interface between RXRα and PPARγ.

**Fig 2:**
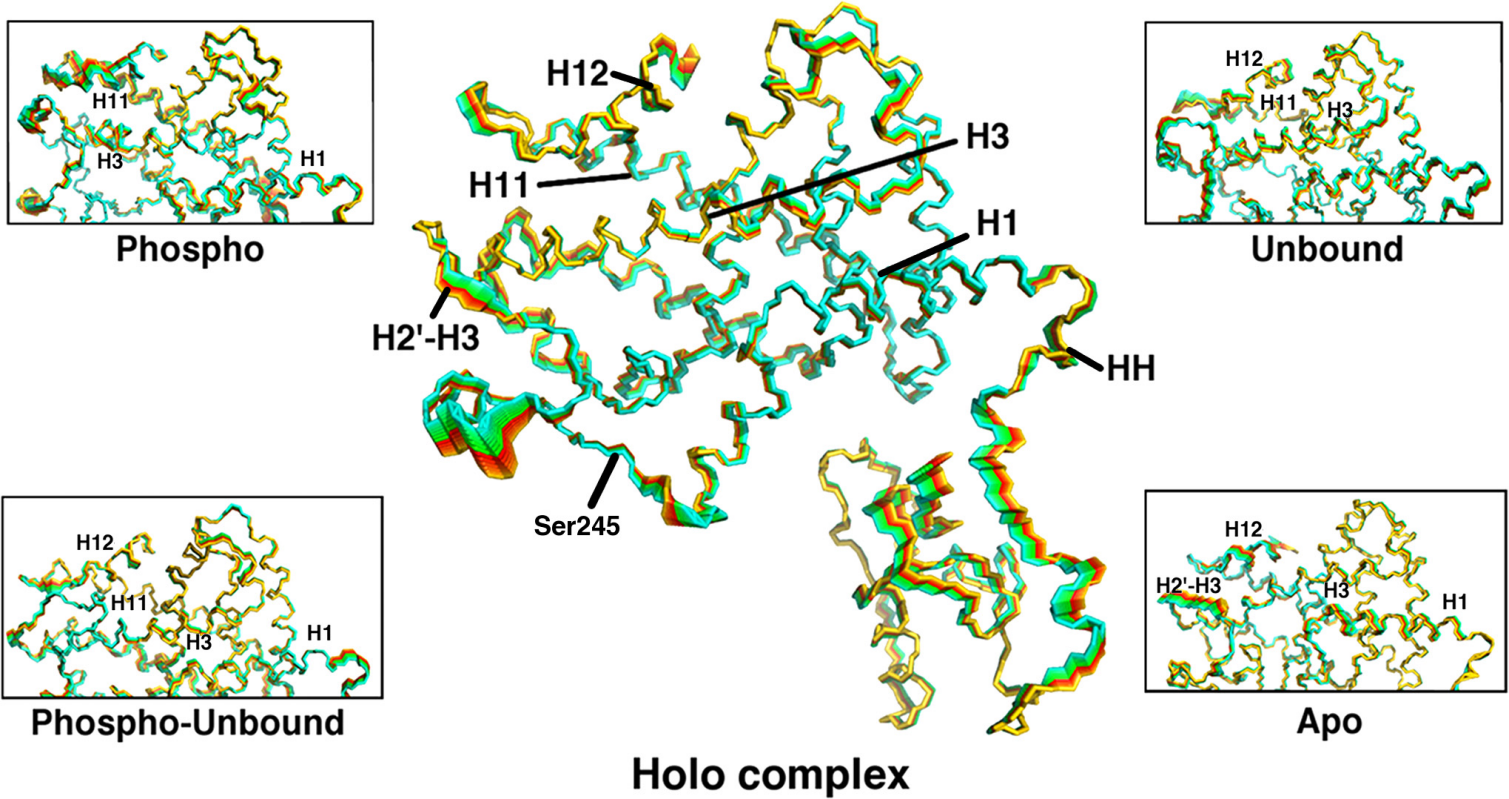
Interpolation of PPARγ structures along the sum of the first 7 eigenvectors from PCA for the holo complex, with insets for each of the other complexes studied here, focusing on the LBD. Cyan indicates completely overlapping regions and thus little or no motion. Red and yellow areas represent those that are more mobile. Critical secondary structure elements are labeled on the holo complex and those features manifesting the greatest movement in each non-holo complex are indicated in the insets.

**Fig 3:**
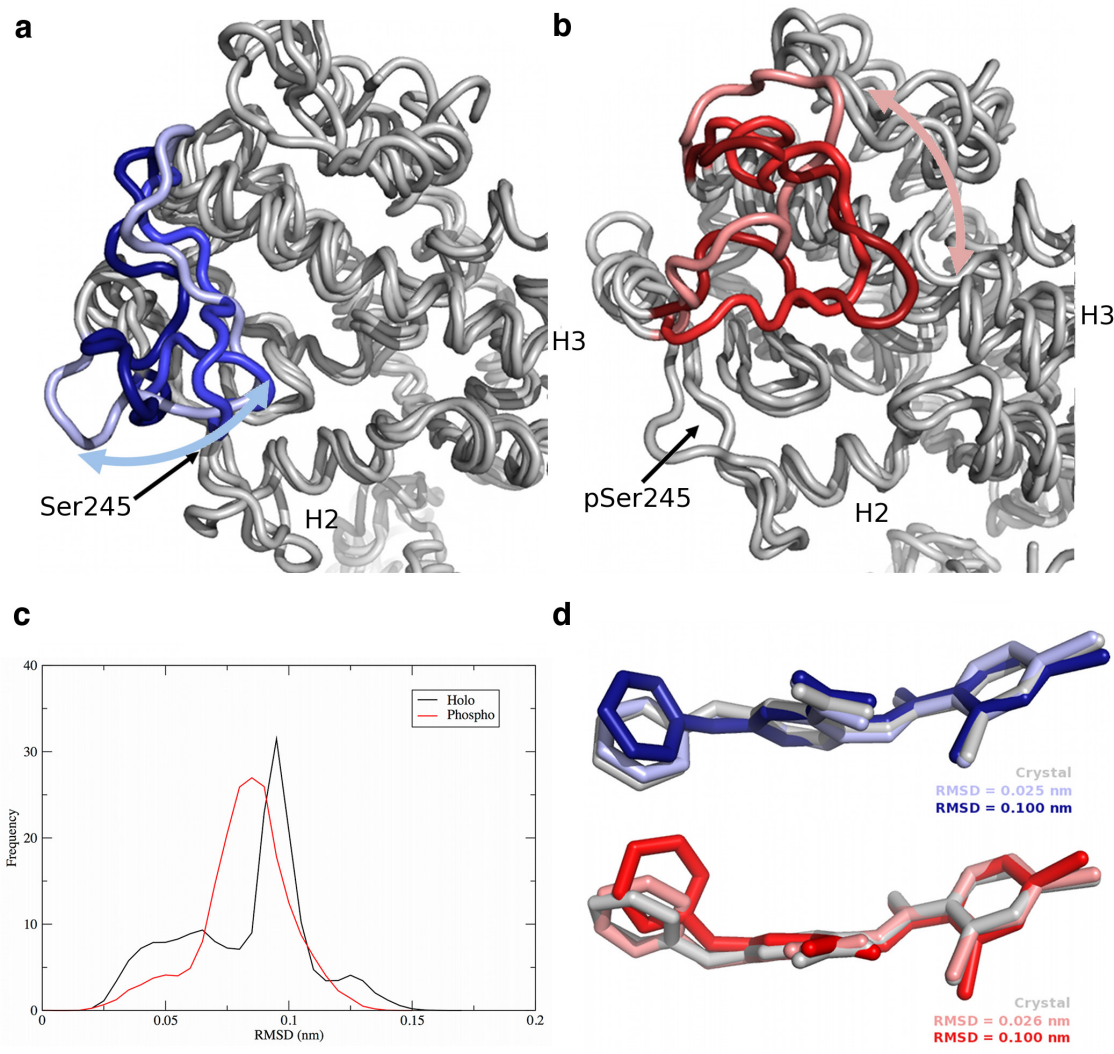
Movement of the H2’-H3 loop and the BVT.13 ligand in PPARγ complexes. Positions of the loop in snapshots from the (a) holo complex and (b) phospho complex (red) simulations, (c) heavy-atom RMSD distributions of the BVT.13 ligand in both the holo and phospho complexes, from data pooled over all frames in all trajectories, and (d) representative conformations of the BVT.13 partial agonist in holo (blue) and phospho (red) complexes. In panels (a) and (b), one structure was taken from each of the three replicate simulations to indicate the three different states (indicated by different shades of red and blue). Helices H2 and H3 are labeled, as is the position of Ser245/pSer245. The “crystal” positions of the ligands in panel (d) are from the energy-minimized, equilibrated structures, with hydrogen atoms removed for clarity.

In the holo complex, the core of the PPARγ LBD remained very rigid (Fig 2). The positions of helices H1, H3, and H11 remained largely invariant over time while flexibility was exhibited in the H2’-H3 loop, the DBD-LBD hinge region, and H12. The H2’-H3 loop moved like a flap, opening and closing over the ligand binding site in the LBD (Fig 3A). In contrast, in the phospho complex, the H2’-H3 loop exhibited a sliding motion, remaining tightly associated with the surface of PPARγ and moving to interact primarily with H3 and the H11-H12 loop (Fig 3B). This sliding motion resulted in increased motion along H1, H3, H11, and H12 relative to the holo complex (Fig 2). The displacement of H12 from its starting position agrees with crystallographic evidence that suggests partial agonism is exerted through H3 and is independent of H12 [8,18]. Helices H3, H11, and H12 form the AF-2 region. Thus increased motion within H3 and H11, as was observed in the case of the phospho complex, can also be postulated to influence coactivator binding. In addition, the H2’-H3 loop interacts with the bound BVT.13 partial agonist in these complexes. In the simulations of the holo complex, BVT.13 adopted two principal conformations, differing in the orientation of the 2-pyrimidinyloxy ring (Fig 3C and 3D). In the holo complex, the interconversion of this ring between the two states occurred on the nanosecond time scale, with the “flipped” orientation (higher heavy-atom RMSD, ~0.10 nm) being sampled approximately 64% of the time. In the phospho complex, BVT.13 was largely locked in an intermediate state at ~0.07 nm and did not interconvert between high and low RMSD states. Given that BVT.13 interacts with the H2’-H3 loop, these differences in conformational sampling can partially explain the differences in loop dynamics.

To analyze the functional implications of phosphorylation, coactivator peptides, and partial agonist BVT.13, we carried out additional simulations of RXRα-PPARγ complexes. The unbound complex, containing the LXXLL coactivator peptide but lacking BVT.13 (Fig 1), behaved much like the phospho complex in terms of the overall motions, with H3 and H11 manifesting increased fluctuation relative to the holo complex. This outcome implies that both phosphorylation and removal of the partial agonist from the ligand-binding site propagate motion along H1, H3, and H11 to destabilize the core of the LBD and the AF-2 region. The phospho-unbound complex showed greater motion throughout H1, H3, and H11 than either the phospho or unbound complexes (Fig 2), indicating that the effects of ligand removal and phosphorylation are additive. The apo complex showed the largest degree of motion throughout the entire LBD (Fig 2), with nearly every helix showing greater flexibility than in any of the other complexes. This outcome is expected, since neither BVT.13 nor the coactivator peptide was bound in the apo complex, thus removing their stabilizing influences. Notably, H3 developed prominent kinks in its structure in the apo structure (S1 Fig), an outcome that would likely disfavor coactivator binding. The presence of BVT.13 in the holo complex stabilized H3, as expected for a partial agonist [8].

In the crystal structure of the ternary complex, Ser245 is in close proximity to Lys145 and Lys201 of the RXRα DBD (S2 Fig). The PPARγ LBD-RXRα DBD interface is also defined by a cluster of hydrophobic residues that may strengthen ionic interactions between pSer245 and these lysine residues. Thus, phosphorylation of Ser245 rigidified the H2-H2’ and H2’-H3 loops, as quantified by backbone root-mean-square fluctuation (RMSF, S3 Fig), causing pSer245 to act as a pivot point within the LBD. Motions within the H2’-H3 loop in the phospho complex were propagated along H1 and H3, displacing them from their crystallographic positions. In the holo complex, the motion of Ser245 was dissipated by H2 and was not as strongly transmitted through H1 and H3 as in the phospho complex, reinforcing the integrity of the LBD and AF-2 region. The implications of this phenomenon on the dynamics of the PPARγ DBD and hinge region will be discussed below.

Chandra et al. identified Phe347 in the PPARγ LBD as a critical residue responsible for interactions with the RXRα DBD that affect DNA binding [4]. In all of our simulations, Phe347 remained tightly associated with the RXRα DBD, thus none of the manipulations performed here negatively impacted the stability of the PPARγ LBD-RXRα DBD interface with respect to Phe347.

### Dynamics of the PPARγ Hinge Region and DBD

In the holo and phospho complexes, the differences in motion manifested in the H2’-H3 loop described above were transmitted across the PPARγ LBD and into the hinge region that connects the DBD and LBD. The hinge interconverted between random coil and helical structures throughout all the simulations. In response to hinge motion, the DBD moved as a rigid body, twisting with respect to the LBD (Fig 2), a phenomenon that was common to all the complexes studied here. The dynamic nature of the hinge region manifested several differences in terms of distance between the hinge and H1, contacts formed between these two regions, and the overall amount of helicity formed in the hinge.

One of the most interesting phenomena that occurred over the course of these simulations was the packing of H1 against residues 194–206 in the hinge region, which frequently formed an α-helix and thus will be referred to here as the “hinge helix” (HH). In the holo complex, the center-of-mass (COM) distance between HH and H1 was 1.6 ± 0.1 nm over the final 400 ns of simulation time, while the same distance in the phospho complex was reduced to 1.4 ± 0.3 nm. By analyzing heavy atom contacts between HH and H1, it can be seen that the hinge packed against the LBD more tightly in the phospho complex than in the holo complex (Table 2) and had reduced flexibility, as measured by RMSF (S3 Fig). By measuring the angle between the principal axes of HH and H1, the interaction between these two helices in the various complexes was further quantified. The phospho complex adopted the narrowest angle between HH and H1 (Table 2). The remaining complexes (apo, unbound, phospho-unbound) adopted intermediate conformations in terms of HH-H1 distances, contacts, and angle between the principal axes. Although these differences are not statistically different, it is notable that the greater flexibility of the LBD of these complexes was propagated throughout the hinge region such that the conformations populated throughout the trajectories were intermediates between holo and phospho forms in terms of HH-H1 interactions using various measures.

**Table 2:** Structural properties of PPARγ*.

Complex	HH—H1 distance (nm)	HH—H1 contacts	HH—H1 angle (degrees)
Holo	1.6 ± 0.2	195 ± 54	65 ± 9
Phospho	1.4 ± 0.3	274 ± 107	49 ± 20
Phospho-Unbound	1.5 ± 0.1	250 ± 40	57 ± 10
Unbound	1.5 ± 0.2	250 ± 45	63 ± 10
Apo	1.6 ± 0.2	214 ± 47	63 ± 17

* Data were averaged over the final 400 ns of three replicate simulations in each set. Values shown are averages and corresponding standard deviations.

**Fig 4:**
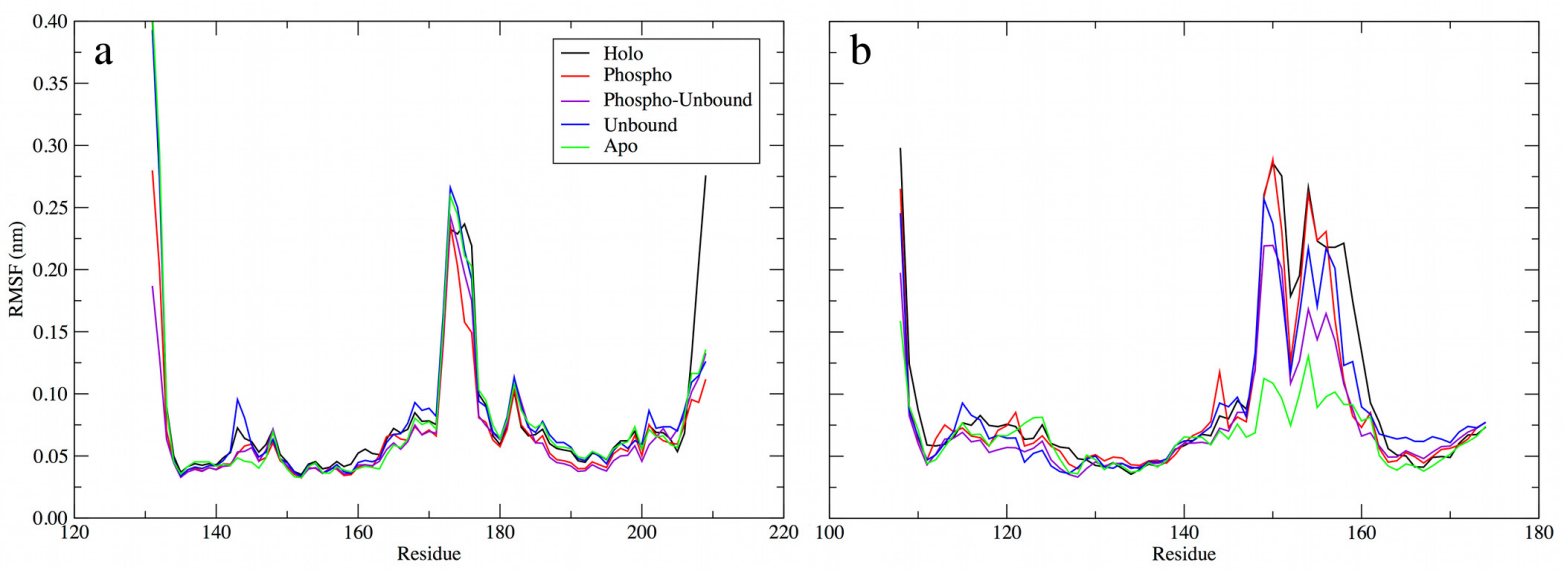
Backbone RMSF of (a) RXRα and (b) PPARγ DBD residues. RMSF was measured from a trajectory pooled from the final 400 ns of each replicate simulation, for a total of 1.2 μs of sampling. A least-squares fit of backbone atoms in each DBD was performed prior to calculating the RMSF.

The dynamics of the hinge region and the phosphorylation state also had implications for the dynamics of the PPARγ DBD. While it is intuitive to expect that the interactions of pSer245 of the PPARγ LBD with the RXRα DBD (S2 Fig) will affect the dynamics of the RXRα DBD, it is not necessarily straightforward to assume that phosphorylation will have any effect on the dynamics of the more distant PPARγ DBD, but our results indicate that such long-range effects exist. We measured the backbone RMSF of the residues in the DBD for both RXRα and PPARγ (Fig 4). Interestingly, phosphorylation of Ser245 had little impact on the dynamics of the RXRα DBD (Fig 4A). While the RMSF of most of the residues in the RXRα DBD was slightly reduced upon phosphorylation of Ser245, the only notable decrease in RMSF occurred in residues 174–179, which reside in a solvent-exposed loop that is not involved in DNA binding and is not near pSer245. Thus, despite its proximity, pSer245 led to no major local perturbations in the dynamics of the RXRα DBD.

Conversely, the PPARγ DBD was perturbed by phosphorylation and/or removal of BVT.13 and coactivator peptides (Fig 4B). While the DBD in the unbound and phospho complexes was only slightly more rigid overall, the phospho-unbound complex DBD was rigidified in a manner that reflects the combined effects of these two modifications. The DBD of the apo complex was dramatically rigidified, to an even greater extent than that of the phospho-unbound complex. Thus, the effect of removing BVT.13 and phosphorylating Ser245 is additive in terms of rigidifying the PPARγ DBD, which may have implications for DNA sequence recognition or binding. Given the changes in structure and dynamics of the LBD in both of these complexes, it is likely that increased fluctuations of the LBD and AF-2 site are compensated by increased rigidity of the DBD. Additionally, in the apo complex, the kinking of helix H3 and concomitant reorientation of the PPARγ DBD relative to the LBD (S1 Fig), perturbed the DBD dynamics such that it packed differently within the complex and became more rigid. pSer245 in the phospho complex led to reduced RMSF in residues 151–153 and 157–160. Asn160 interacts directly with the PPRE sequence, in the minor groove of the bound DNA [4]. All of these observations suggest that long-range motions, communicated from the RXRα DBD-PPARγ LBD interface (the location of phosphorylation), through the PPARγ LBD (including the ligand-binding site and helix H3), affect the dynamics of the RXRα LBD-PPARγ DBD interface and the PPARγ DBD itself. While the implications for DNA binding and stability remain to be fully understood, the fact that small, distant changes give rise to this phenomenon suggests a large degree of cooperativity within the PPARγ-RXRα-DNA complex.

### Dynamics of the LBD-LBD Interface

**Fig 5:**
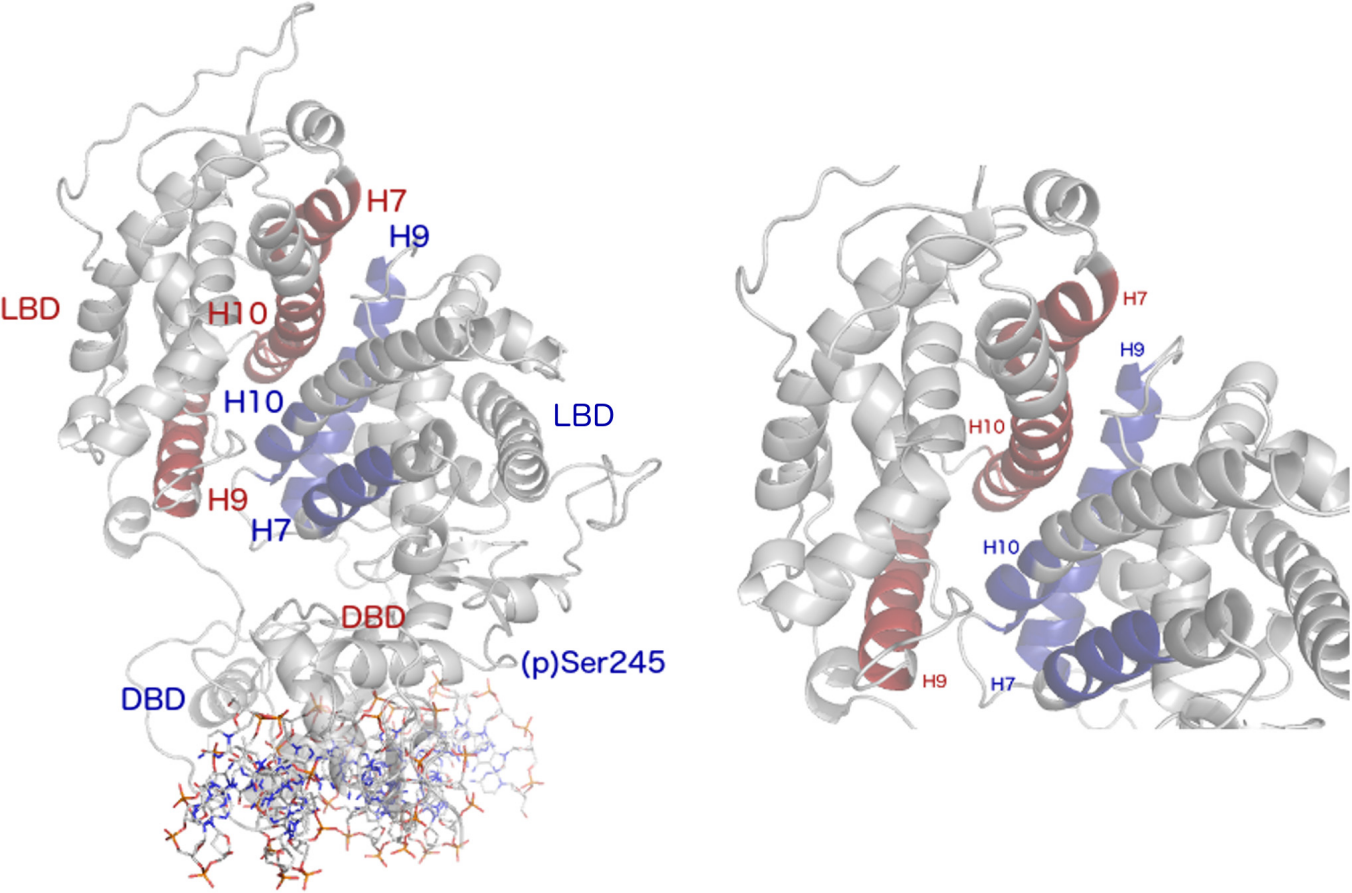
The PPARγ-RXRα LBD-LBD interface. Labels in blue correspond to structural features of PPARγ, while those in red correspond to RXRα. At left is the full complex, including the PPRE DNA sequence. At right is a zoomed-in view of the LBD-LBD interface.

There are three main interfaces for protein-protein interactions within the PPARγ-RXRα complex; the LBD-LBD, the PPARγ LBD-RXRα DBD, and the RXRα LBD-PPARγ DBD. Given the large surface area of the LBD-LBD interface, encompassing helices H7, H9, and H10 of each protein (Fig 5) [4], it is reasonable to expect that LBD-LBD dynamics contribute to communication across the ternary complex, especially given the observations regarding the PPARγ DBD described above. To characterize the dynamics of the LBD-LBD interface, we monitored the number of heavy atom (non-hydrogen) contacts maintained over time and performed PCA on the interfacial helices.

The holo complex maintained the fewest heavy atom contacts (407 ± 58) over the final 400 ns of simulation time. The data set produced a narrow distribution (S4 Fig), indicating homogeneity in the sampling. Contacts were increased in the remaining complexes, with 442 ± 63 contacts persisting in the phospho complex, and the apo and unbound complexes maintaining the largest number of contacts, with 451 ± 92 and 452 ± 60, respectively. In the phospho-unbound complex, 424 ± 62 contacts persisted. We also note that the apo complex produced a bimodal distribution, with a considerable population centered at approximately 600 contacts, indicating a very tight interaction. While the differences in numbers of contacts are not significantly different, the results lead us to propose that proper function of the RXRα-PPARγ complex requires plasticity at the LBD-LBD interface, and that phosphorylation of Ser245 or the absence of BVT.13 and/or coactivators modulates these dynamics by altering this plasticity.

We further characterized the dynamics of the LBD-LBD interface by conducting PCA (see Methods). First, the different motions of each complex were characterized, as differences emerged based on phosphorylation state and presence or absence of ligands and coactivator peptides. Next, the conformational overlap of the non-holo (phospho, apo, unbound, and phospho-unbound) complexes with the holo complex was assessed by projecting non-holo conformations onto the eigenvectors derived from the holo complex simulations.

If the LBD-LBD interface is visualized as a plane between the two protein subunits, the motion along the first eigenvector in the holo complex was scissoring between the two LBDs, out of the plane of this interface (S5 Fig). The H10 helices of each protein formed a rigid pivot point. While H7 and the N-terminal end of H9 in PPARγ approached the C-terminal end of H9 in RXRα, the same helices in RXRα moved in opposition. The net effect was an opening at one end of the interface, concomitant with a closing at the other end. A kink developed in RXRα H9 as part of its progression across this eigenvector. The scissoring motion was coupled with a slight twisting motion between the two domains (the second eigenvector) in the plane of the dimerization interface such RXRα and PPARγ rotated in opposite directions over time.

The interfacial dynamics of the non-holo complexes (S6–S9 Figs) showed subtle differences from the holo complex. In the phospho complex (S6 Fig), the magnitude of the scissor motion was much larger than in the case of the holo complex. Whereas the C-terminal residues of RXRα H9 adopted a kinked structure in the holo complex, the N-terminal residues of p-PPARγ H9 kinked, instead, likely due to a rigidifying effect of the ionic interactions between the p-PPARγ LBD and the RXRα DBD due to pSer245 (S2 Fig). The primary mode of LBD-LBD motion in the apo complex was twisting around H10, concomitant with in-plane rotation. The second eigenvector represented a sliding motion between the two LBD. Both the unbound and phospho-unbound complexes (S8 and S9 Figs, respectively) exhibited twisting between the two LBD as the principal mode of motion. In the unbound complex, a kink formed in RXRα H7. In the phospho-unbound complex, the N-terminal residues of PPARγ H9 and RXRα H7 underwent scissor-like motion like the phospho complexes (S9 Fig). Thus, the phospho-unbound complex resembled both the phospho and unbound complexes.

**Fig 6:**
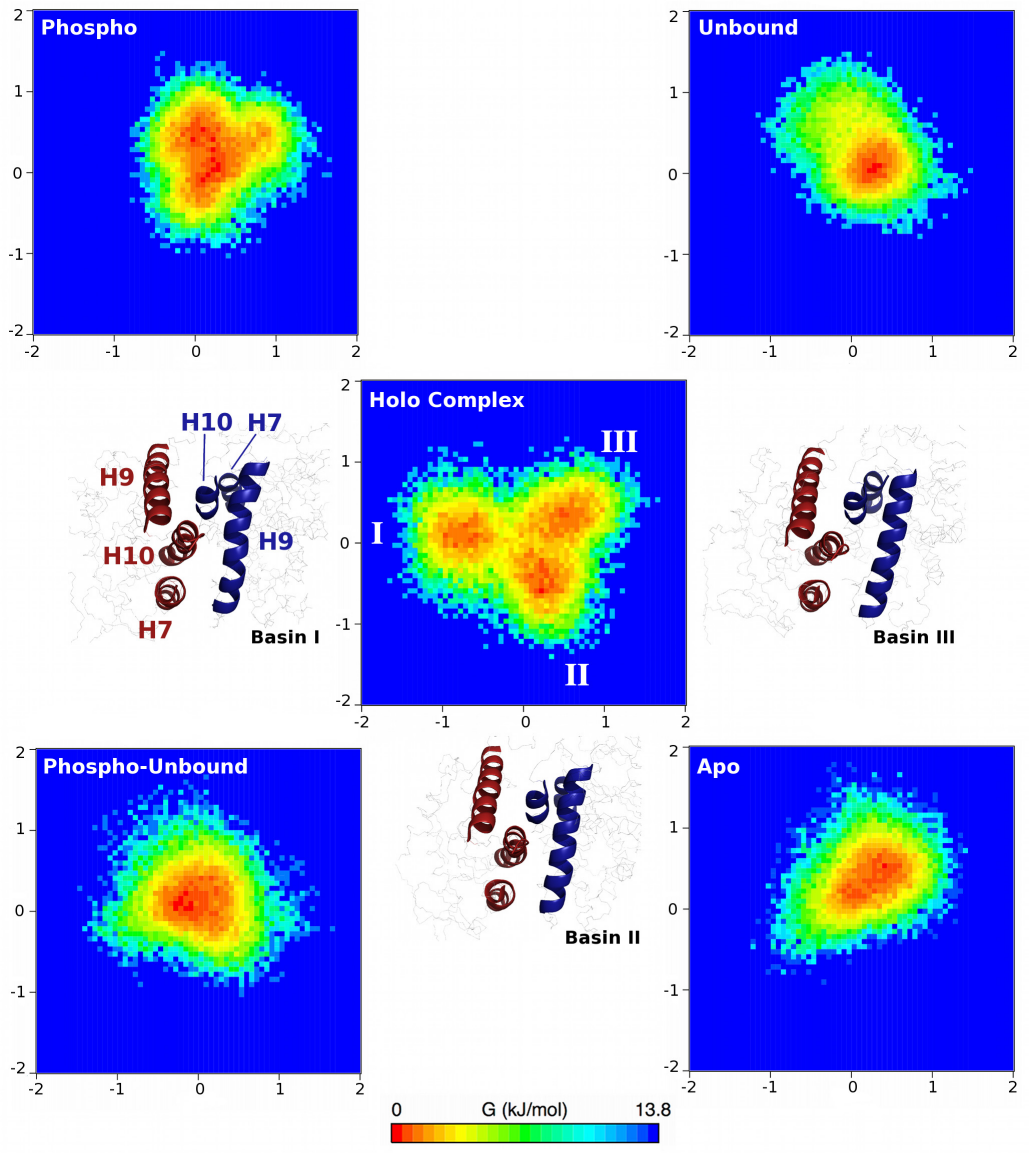
Free energy surfaces of interfacial dynamics of all complexes studied here. Three free energy minima are identified in the holo complex simulations, and are labeled by Roman numerals. Images corresponding to representative holo complex conformations of each basin are shown, with the conformations being positioned most closely to the respective basins to which they correspond. Interfacial PPARγ and RXRα helices are in blue and red, respectively, and are labeled in the image nearest to Basin I. Positions along eigenvector 1 (x-axis) and eigenvector 2 (y-axis) are shown in nm for each free energy surface.

The findings from the interfacial PCA lead us to propose that the coupling of scissor-like and twisting motions of the holo complex is indicative of transcriptional activity. That is, the communication from one side of the complex (the RXRα DBD) to the other (the PPARγ DBD) is mediated by the dynamics of the LBD-LBD interface, which may also be influenced by the presence of bound ligands and coactivators. The non-holo complexes exhibited altered dynamics that correspond to non-functional modes. The phospho complex behaved similarly to the holo complex, though the magnitude of these functional motions was diminished, indicating that binding of a partial agonist such as BVT.13 partially recovers functional dynamics, in agreement with experimental findings [15]. For a more quantitative comparison between these complexes and to visualize the phase space differences between the complexes, the conformations of all of the non-holo complexes were projected onto the eigenvectors of the holo complex, with the probabilities of (λ_1_, λ_2_) combinations used to produce free energy surfaces (Fig 6, see equation in Methods).

**Fig 7:**
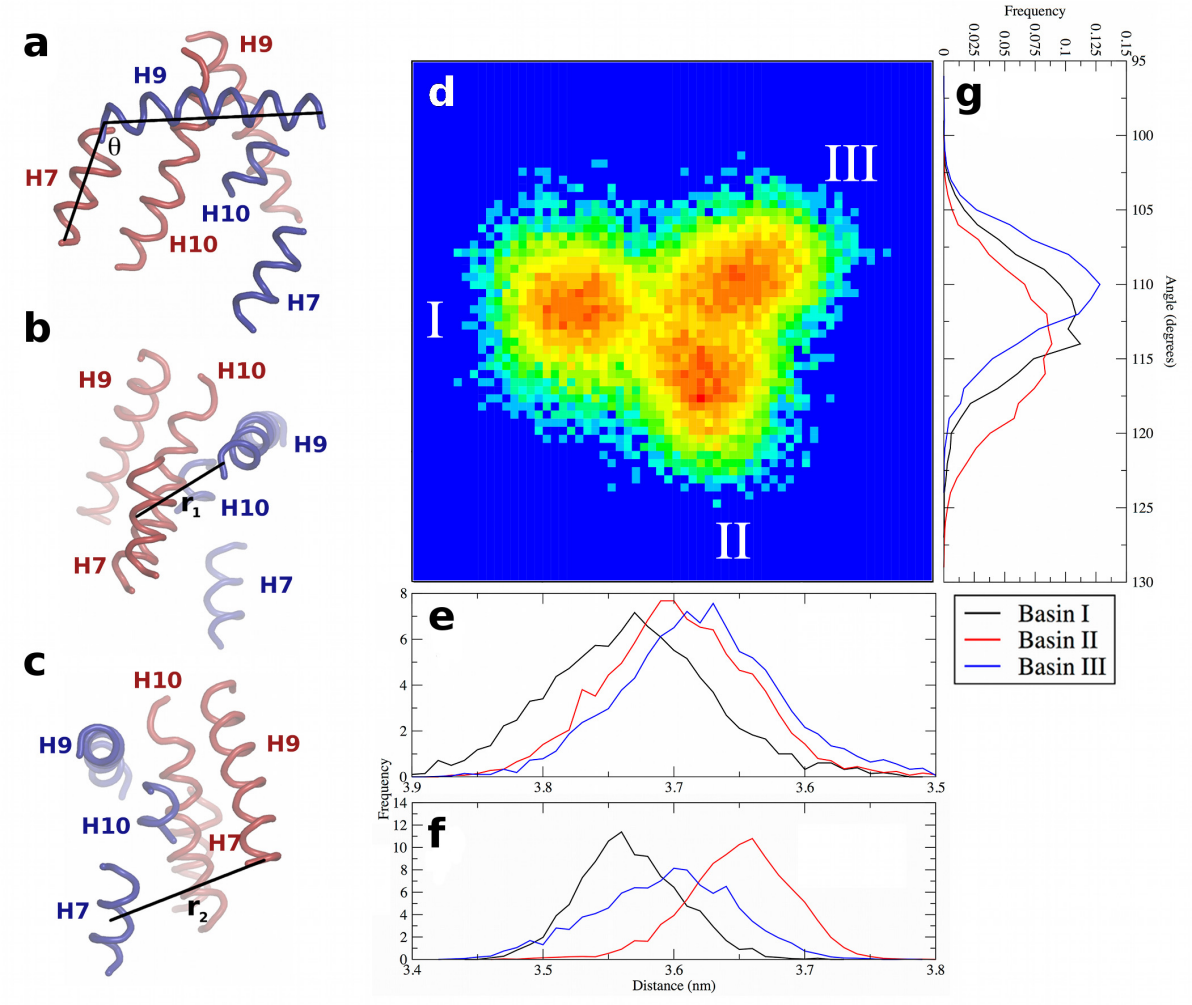
Characterization of PPARγ-RXRα tertiary structure dynamics at the LBD-LBD interface of the holo complex. (a) The twist angle θ between the two subunits, and distances (b) r_1_ and (c) r_2_. In panels (a—c), structural elements of PPARγ and RXRα are in blue and red, respectively, and are labeled in each panel to indicate the relative orientation. Panel (d) is the free energy surface from PCA, enlarged from Fig 6, with basins labeled. Panels (e) and (f) show the distributions of r_2_ and r_1_, respectively, in all three basins. Panel (g) shows the distributions of θ in all three basins. Note the x-axes in panels (e) and (f) are reversed to more clearly correspond to the properties of the basins, as described in the text. The legend in the bottom-right referring to the basins is applicable to panels (e—g).

There are three minima in the free energy surfaces for the holo complex simulations, labeled Basins I, II, and III (Fig 6). Visualization of the trajectories indicates that the holo complex cycles through Basins II, I, and III before returning to Basin II. The basins are separated by barriers of no larger than 3.5 kJ mol^-1^, thus they can be readily crossed at physiological temperature. The changes in tertiary structure that comprise the configurations in these basins can be quantified in terms of a twisting angle (θ) and two distances (r_1_ and r_2_) as shown in Fig 7. The twisting angle is that formed by a vector drawn through H9 of PPARγ from N- to C-terminal ends, and from the C-terminal end of PPARγ H9 to the C-terminal end of RXRα H7 (Fig 7A). The two distances measure the extent to which either end of the interface is “open” or “closed,” with r_1_ being the distance from the COM of Asn424 in PPARγ to the COM of RXRα H7 (Fig 7B) and r_2_ being the distance from the COM of the Lys407 in RXRα to the COM of PPARγ H7 (Fig 7C). By measuring each of these quantities in the three basins (Fig 7E, 7F and 7G), it is possible to illustrate how the structural changes are mapped onto the free energy surface from PCA (Fig 7D, identical to the central image in Fig 6). Though there is considerable overlap in these quantities, particularly distances r_1_ and r_2_, the combined effects of simultaneous twisting and scissoring can characterize the configurations in each basin.

Basin I is characterized by an intermediate value of θ (112 ± 4°) and r_1_ at its minimum (3.57 ± 0.04 nm) and r_2_ at its maximum (3.73 ± 0.06 nm). Thus, in Basin I, the effects of scissoring motions are at their maximum, and the RXRα H9—PPARγ H7 end of the interface is “open” while the PPARγ H9—RXRα H7 end is “closed.” In Basin II, θ is at its maximum (114 ± 4°) and r_2_ (3.70 ± 0.06 nm) remains comparable to the value found in Basin I, while r_1_ increases to 3.65 ± 0.04 nm. Thus an increase in rotation between the two subunits in structures taken from Basin I to Basin II begins to open the previously “closed” end of the interface. In Basin III, θ is at its minimum (110 ± 3θ), indicating the least twisted of the three basins. In Basin III, r_1_ is at an intermediate value of 3.60 ± 0.05 nm, while r_2_ is nearly unchanged (3.68 ± 0.06 nm) relative to Basin II. As a result, the transition between Basin II and Basin III can be described as a relaxation in the twist angle with a slight closing along r_1_. Further closing along r_1_ and a slight increase in twist yields configurations populating Basin I. Thus, the transitions between any two basins can be described by 2–4° of twist between the LBD and opening or closing along one of the two characteristic distances at either end of the interface.

The other complexes populated conformations that reflected only portions of the holo complex free energy surface. The phospho complex (Fig 6, top left) populates Basins II and III, but does not populate Basin I and heavily samples conformations in excess of +1.0 nm along eigenvector 2 (twisting motion), a region not sampled at all in the holo complex. The phospho-unbound complex (Fig 6, bottom left) samples conformations that do not belong discretely to any of the identified Basins, instead remaining trapped in the middle of the free energy surface. Thus, the binding of BVT.13 allows the phospho complex to sample some holo-like conformations that are absent even when the coactivator peptides are bound. The apo complex (Fig 6, bottom right) only sparsely populates Basins I and III, and its free energy minimum is located between those two basins and towards the positive extreme of eigenvector 2, similar to the sampling of the phospho complex. If the apo form of the complex is considered to be an inactive complex, this finding indicates that the phospho complex populates inactive conformations, accounting for its reduced activity compared to the holo complex. Finally, the unbound complex (Fig 6, top right) shows narrow sampling along the holo complex eigenvectors, sampling conformations between Basins II and III, but belonging strongly to neither. The unbound complex also sampled apo-like conformations along eigenvector 2. Given that all of the non-holo complexes sampled large eigenvalues in the positive direction along eigenvector 2, we conclude that the presence of the BVT.13 partial agonist and the absence of phosphorylation on Ser245 limit the twisting motion at the LBD-LBD interface. The PCA results can be further interpreted in light of the interfacial contacts described above. The holo complex maintained the fewest interfacial contacts over time, allowing conformational freedom to sample all three Basins. The apo complex sampled conformations with dramatically increased contacts, consistent with a more rigid interface that does not populate functional states. The other complexes had intermediate plasticity, reflected in their occupancy of relatively narrow regions of the holo complex free energy surface (Fig 6).

### Dynamics of the PPARγ AF-2 Site

The structure and dynamics of the PPARγ AF-2 region have implications for the ability of PPARγ to bind coactivator proteins that dictate its transcriptional activity. Unlike the holo complex, the phospho complex showed displacements propagated along H1, H3, the H11-H12 loop, and H12, leading to greater mobility within the AF-2 region (Fig 2). As shown in Fig 3, phosphorylation caused the H2’-H3 loop to behave differently than in the holo complex such that it moved to interact with the H11-H12 loop significantly more extensively (175 ± 52 heavy atom contacts over the final 400 ns compared to 36 ± 7 in the case of the holo complex). These interactions propagated forces to H12 in the AF-2 region, leading to destabilization. Thus, though the coactivator peptide remained bound during the simulations conducted here, the AF-2 region was more dynamic due to pSer245.

The AF-2 region can be characterized in part by a “charge clamp” formed by Lys301 and Glu471 [19–21], which form hydrogen bonds with the coactivator peptide to coordinate its binding to PPARγ. In the holo complex, the Lys301-Glu471 distance was 1.81 ± 0.09 nm over the final 400 ns of simulation time. This distance was slightly increased in the phospho complex, 1.89 ± 0.07 nm. The charge clamps in the unbound and apo complexes were separated by 2.0 ± 0.3 nm and 2.0 ± 0.2 nm, respectively, the longest distances of any of the complexes. In the phospho-unbound complex, the distance was 1.83 ± 0.09 nm, nearly identical to that of the phospho and holo complexes. Thus, we conclude that phosphorylation of Ser245, though it propagates motion through H3, does little to destabilize the Lys301-Glu471 charge clamp, though removal of BVT.13 and/or the coactivator does destabilize it.

## Discussion

In the present work, we have carried out extensive simulations of ternary RXRα-PPARγ1-DNA complexes, examining the influence of phosphorylation, ligands, and coactivators on collective motions. We have collected 1.5 μs of sampling for each complex, representing the most complete assessment of PPARγ dynamics using MD simulations to date. The experimental finding that pSer112 can shift the conformational ensemble of ligand-free PPARγ LBD [14] motivated the present work. Since the structure of the A/B domain has not been determined, we focused on phosphorylation within the PPARγ LBD. Since phosphorylation in a domain spatially distant from the LBD influences the dynamics of the LBD and ligand binding, we set out to determine if phosphorylation within the LBD would also impact functional dynamics.

It is important to recognize that some disagreement exists regarding the structure of the PPARγ-RXRα-DNA structure in solution. While the current work makes use of the only atomic-resolution structure that is available, work on the same structure in solution suggests that the complex adopts a more elongated conformation [22–24]. In solution, scattering data indicate that the PPARγΔNTD-RXRαΔNTD-DR1 complex (with N-terminal A/B domains deleted from each protein, the same complex examined here) adopts conformations with radius of gyration (R_g_) values of approximately 37–39 Å [22,23]. In our simulations of the holo complex, the average R_g_ value was 30.2 ± 0.3 Å. Thus, it is clear that there are small differences between these structures, with the crystal structure giving rise to slightly more compact conformations than experimental measurements in solution. Given that detailed atomic-resolution structures from solution experiments are unavailable, the crystal structure utilized in this work is the most appropriate for approaching the problems at hand, though it is important to note the caveat that dynamics in solution may, in fact, be somewhat different. Additional experimental and theoretical work will be needed to further investigate these issues.

It also has been suggested that the use of static structures to assess dynamic systems and relationships can prove problematic in elucidating the details of the PPARγ-small molecule binding process [25]. The use of MD simulations is useful in these cases because it sheds light on theories regarding events that occur on time and length scales that are inaccessible to most experimental methods. We do not attempt to resolve the differences seen between static (crystal) structures, which can be influenced heavily by the conditions of the crystallization and data collection process. Instead, we aim to provide detailed insights into the larger puzzle that may help drive experimental efforts to resolve outstanding questions.

Another consideration in conducting this work is the relevance of the BVT.13-bound p-PPARγ complex. We recognize that BVT.13 and related compounds (e.g., MRL24) inhibit Cdk5-mediated phosphorylation of PPARγ, though as noted by Choi et al. [15], it is likely that binding of these ligands results in a mixed population of phosphorylated and non-phosphorylated forms. In addition, in a group of patients treated with rosiglitazone, which also inhibits phosphorylation of Ser245, most, but not all, had a decreased level of phosphorylation [15]. These observations suggest that multiple populations, which vary in terms of bound ligand and phosphorylation, will exist. Thus, the systems studied here, including the phosphorylated, ligand-bound form of PPARγ, are relevant and are appropriate for comparison.

Phosphorylation affects the dynamics of the H2’-H3 loop and the conformational sampling of the BVT.13 partial agonist within the ligand-binding site. The partial agonist BVT.13 sampled two conformations, with the subpopulations dependent on the phosphorylation state of PPARγ. This outcome agrees with observations made by Hughes et al. regarding their work with partial agonists MRL20 and MRL24 [25], though the effects observed in the present work are less dramatic in terms of differences in structure of the BVT.13 conformers. Despite the structural similarity between BVT.13 and MRL24, these two ligands show different propensities to reorient within the PPARγ binding site or bind in different conformations. The issue of multiple possible binding modes versus active dynamic motion repositioning the ligand after the initial binding event was not resolved in their work [25]. Our simulations suggest BVT.13 can bind in one conformation but does not remain locked in this conformation. Such switching could alter the conformational sampling of PPARγ or potentially change the recruitment activity from one coactivator to another as a way to drive multiple gene regulation events. The change in binding mode also appears to be affected by phosphorylation, which would indicate this posttranslational modification plays a significant role in the specificity of the gene regulation process.

Our results further suggest that phosphorylation of Ser245 (Ser273 in PPARγ2) has far-reaching implications for the dynamics of PPARγ and the complex as a whole. The pSer245 residue interacted very tightly with Lys145 of the RXRα DBD, quenching the motions of neighboring residues while leading to greater fluctuations in the positions of nearby helices H1 and H3. The result is that the LBD of the phospho complex, including the AF-2 region, is more dynamic than in the case of the holo complex. Additionally, phosphorylation affected the PPARγ-RXRα LBD-LBD interface, rigidifying it and perturbing the twisting and scissoring motions that were revealed by PCA in the case of the holo complex. Our data suggest that in the holo PPARγ complex, the LBD-LBD interface cycles between three conformational states, unlike the other complexes examined here, which occupy narrower regions of the holo complex free energy surface, or different regions altogether (Fig 6). The phosphorylated complex sampled a narrower conformational ensemble, including some states similar to the inactive apo complex. These findings agree with a proposal of Bruning et al. [26] that the gradient of PPARγ transactivation by ligand binding is not solely dependent upon changes in the AF-2 region. Instead, they proposed that allosteric or other long-range dynamics contribute to these effects, also suggesting a possible role for RXRα conformational sampling in this process. Our results, the first to provide atomic-resolution insight into this process, suggest that indeed such long-range allostery is at play, and that the LBD-LBD interface is dramatically impacted by phosphorylation and/or ligand binding.

We observed that phosphorylation of Ser245 had long-range effects on the dynamics of the PPARγ hinge region and DBD. Rather than affect local interactions with the RXRα DBD, pSer245 exerted its effects across the complex and rigidified the PPARγ DBD, especially Asn160, which binds the PPRE. The RMSF of the apo complex DBD was dramatically reduced, which we interpret as an indicator of reduced activity, given that the apo structure should be the least active of all of those studied here. If this is the case, the fact that a key DNA-binding sequence in the PPARγ DBD was rigidified may explain the experimental observation that p-PPARγ modulates the expression of different genes than does the holo complex [15], that is, there may be some fundamental differences in the dynamics of protein-DNA interactions that alter gene expression. Previous work on the vitamin D receptor-RXRα-DNA [27] and glucocorticoid receptor-DNA [28] complexes suggests that DNA binding impacts the conformational dynamics of the respective LBD. Thus, the function of nuclear hormone receptors appears to be dependent upon long-range communication between multiple protein domains and interactions with DNA.

Our results indicate that phosphorylation of Ser245 at the PPARγ LBD-RXRα DBD interface has far-reaching consequences for the dynamics of distant parts of the ternary complex, highlighting the importance of collective inter- and intra-domain motions in functional dynamics. MD simulations of the RXRα LBD found that phosphorylation caused local reorganization of salt bridges, leading to changes in the coactivator-binding site on the opposite face of the LBD [29]. In conjunction with our results, these findings suggest that changes in global dynamics as a result of phosphorylation may be a general feature of nuclear hormone receptors. Similarly, MD simulations also found that ligand binding influences a complex network of collective motions within the PPARγ LBD [30]. Our study is the first to demonstrate the role of inter-protein cooperative motions within the full PPARγ-RXRα-DNA complex. Finally, our results suggest that partial agonists likely partially recover activity in phosphorylated PPARγ by (i) stabilizing AF-2, specifically helices H3 and H11 that are otherwise perturbed by phosphorylation and (ii) promoting the occupancy of some holo-like states of the PPARγ-RXRα LBD-LBD interface. These results confirm the importance of inter-domain interactions and dynamics in the proper functioning of the RXRα-PPARγ-DNA ternary complex, as suggested by Chandra et al. [4].

## Methods

The structure of the PPARγ-RXRα-DNA complex was taken from PDB entry 3DZU [4]. The structure contains PPARγ isoform 1, RXRα, LXXLL coactivator peptides (fragments of the larger proteins found *in vivo*) bound to both PPARγ and RXRα, retinoic acid bound to RXRα, and partial agonist BVT.13 bound to PPARγ. The heterodimeric protein complex is bound to the PPAR response element (PPRE). Missing loops in the protein structures were reconstructed with the ModLoop server (http://modbase.compbio.ucsf.edu/modloop/) [31,32]. This ternary complex is referred to as the holo complex. The phospho complex was prepared by adding a phosphate group to Ser245 of PPARγ (corresponding to Ser273 in PPARγ2 in the work of Choi et al. [15,16]) in the xLeap module of AmberTools (www.ambermd.org). Removal of retinoic acid and partial agonist BVT.13, but retention of coactivator peptides, from the holo complex yielded the unbound complex. Removal of retinoic acid, BVT.13, and coactivator peptides from the holo complex yielded the apo complex. The BVT.13 ligand was removed from PPARγ in the phospho complex (while retinoic acid was retained in RXRα) to yield the phospho-unbound complex. These complexes (summarized in Table 1) were constructed to elucidate the role of each component (phosphorylation, ligands, and coactivator peptides) in the underlying dynamics of the PPARγ-RXRα-DNA complex.

The protein and DNA components of each complex were assigned parameters from the AMBER99SB-ILDN force field [33], and ligand (retinoic acid and BVT.13) parameters were generated using GAFF [34]. Ligand topologies for use in GROMACS are provided in S1 Table. Each complex was centered in a rhombic dodecahedral simulation cell with a minimum box-solute distance of 1.0 nm. The unit cell was then filled with TIP3P water [35] and ~150 mM NaCl, in addition to Na^+^ counterions sufficient to neutralize the net charge on each complex. All ionizable amino acids were assigned their dominant protonation state at pH 7.4 according to pK_a_ predictions by the H++ server (http://biophysics.cs.vt.edu/H++) [36–38], except the cysteine residues that coordinate Zn^2+^ ions in each DBD; these residues were treated as anionic, as predicted by quantum mechanical calculations [39].

Simulations were carried out with GROMACS [40,41], version 4.6. All bonds were constrained using the P-LINCS algorithm [42], allowing an integration time step of 2 fs. The Verlet cutoff scheme [43] was used with a minimum cutoff of 1.0 nm for short-range Lennard-Jones interactions and the real-space contribution to the smooth Particle Mesh Ewald algorithm [44,45], which was used to compute long-range electrostatic interactions. Dispersion correction was applied to energy and pressure terms and periodic boundary conditions were applied in all three dimensions.

Each system was equilibrated in two phases during which restraints were placed on protein and DNA heavy atoms, first under an NVT ensemble for 100 ps using a weak coupling algorithm with stochastic rescaling [46] to maintain temperature at 310 K. NVT equilibration was followed by NPT equilibration for 500 ps using the same thermostat and the Parrinello-Rahman barostat [47,48] to maintain pressure at 1 bar. Production simulations were carried out under an NPT ensemble in the absence of any restraints. Three independent simulations of 500 ns in length were conducted for a total of 1.5 μs of sampling for each complex. Convergence of each trajectory was assessed by monitoring backbone root-mean-square deviation (RMSD) of each protein over time (S10 and S11 Figs). Backbone RMSD values were generally stable after the first 100 ns of each simulation. Analysis was carried out using programs within the GROMACS package. For PCA, snapshots from the final 400 ns of each trajectory were pooled to create a single “trajectory” representing 1.2 μs of sampling for each complex. Doing so guarantees that the eigenvectors identified and resulting free energy surfaces were representative of all three replicate simulations. To further assess convergence, PCA was performed again over the final 250 ns of each trajectory. Given that the same motions were observed and the same regions of phase space sampled, the results from the final 400 ns were used for the most complete and statistically rigorous analysis possible. PCA was carried out on each protein, first by fitting to backbone atoms in the protein to remove global rotational and translational modes. For interfacial PCA, structures were first fitted to the backbone of the helices considered in the analysis (H7, H9, and H10) before diagonalizing the covariance matrix. Free energy surfaces were constructed from the PCA eigenvector plots using a two-dimensional histogram approach, according to the equation

$$\Delta G\left(\lambda_{1},\lambda_{2}\right)=-k_{B}T\left[\ln\left(\lambda_{1},\lambda_{2}\right)-\ln P_{max}\right]$$

in which λ_1_ and λ_2_ are the eigenvalues of the first and second eigenvectors, and P_max_ is the (λ_1_,λ_2_) combination that occurs with the greatest frequency, thus defining the energy minimum of the surface. These free energies are not absolute or conformational free energies, as no enthalpic or entropic terms have been calculated.

## Supporting Information

S1 Fig Comparison of representative H3 conformations from the holo and apo complexes. For clarity, only PPARγ is shown in a cartoon representation. H3 is shown in blue to better illustrate its conformation.
(TIF)

S2 Fig The local environment around pSer245. Shown in sticks are pSer245 of PPARγ (blue) as well as Lys145 and Lys201 of RXRα (gray). Hydrophobic residues of RXRα that define the interaction interface between the PPARγ LBD and the RXRα DBD are shown as gray spheres. The double-stranded DNA molecule is shown as a cartoon.
(TIF)

S3 Fig Backbone root-mean-square fluctuation (RMSF) of PPARγ for holo and phospho complexes after fitting to backbone atoms in PPARγ. RMSF values for residues flanking pSer245 in the phospho complex are notably reduced relative to Ser245 in the holo complex. The most prominent structural features are labeled. DBD = DNA-binding domain, and helices are denoted as HH (“hinge helix,” see main text), H1, H3, etc.
(TIF)

S4 Fig Probability distributions of interfacial heavy atom contacts for all five complexes. (TIF)

S5 Fig Motions along eigenvector 1 for the holo complex simulations. The top panel shows a cartoon of the main scissor and twist motions described in the main text, with PPARγ in blue, RXRα in red, and DNA represented as a tan cylinder. In the bottom panel, PPARγ helices are shown in blue (dark and light representing extremes along the eigenvector), while RXRα helices are shown in red (dark and light again representing extremes). Each helix is labeled, with blue and red indicating PPARγ and RXRα, respectively. The left and right panels are different views, rotated around the vertical axis between the PPARγ and RXRα LBD. H7 of RXRα and H9 of PPARγ “open” while H9 of RXRα and H7 of PPARγ “close,” and vice versa. Arrows indicate the eigenvalues along the eigenvector, with motions only illustrated if they were larger than 0.2 nm.
(TIF)

S6 Fig Motions along eigenvector 1 for the phospho complex simulations. PPARγ helices are shown in blue (dark and light representing extremes along the eigenvector), while RXRα helices are shown in red (dark and light again representing extremes). Each helix is labeled, with blue and red indicating PPARγ and RXRα, respectively. The left and right panels are different views, rotated around the vertical axis between the PPARγ and RXRα LBD. H7 of RXRα and H9 of PPARγ “close” while H9 of RXRα and H7 of PPARγ “open,” though the opening motion is muted compared to the holo complex, and vice versa. Arrows indicate the eigenvalues along the eigenvector, with motions only illustrated if they were larger than 0.2 nm.
(TIF)

S7 Fig Motions along eigenvector 1 for the apo complex simulations. PPARγ helices are shown in blue (dark and light representing extremes along the eigenvector), while RXRα helices are shown in red (dark and light again representing extremes). Each helix is labeled, with blue and red indicating PPARγ and RXRα, respectively. The left and right panels are different views, rotated around the vertical axis between the PPARγ and RXRα LBD. Arrows indicate the eigenvalues along the eigenvector, with motions only illustrated if they were larger than 0.2 nm.
(TIF)

S8 Fig Motions along eigenvector 1 for the unbound complex simulations. PPARγ helices are shown in blue (dark and light representing extremes along the eigenvector), while RXRα helices are shown in red (dark and light again representing extremes). Each helix is labeled, with blue and red indicating PPARγ and RXRα, respectively. The left and right panels are different views, rotated around the vertical axis between the PPARγ and RXRα LBD. Arrows indicate the eigenvalues along the eigenvector, with motions only illustrated if they were larger than 0.2 nm.
(TIF)

S9 Fig Motions along eigenvector 1 for the phospho-unbound complex simulations. PPARγ helices are shown in blue (dark and light representing extremes along the eigenvector), while RXRα helices are shown in red (dark and light again representing extremes). Each helix is labeled, with blue and red indicating PPARγ and RXRα, respectively. The left and right panels are different views, rotated around the vertical axis between the PPARγ and RXRα LBD. Arrows indicate the eigenvalues along the eigenvector, with motions only illustrated if they were larger than 0.2 nm.
(TIF)

S10 Fig Backbone RMSD time series for both PPARγ and RXRα. The RMSD for each protein was calculated after performing a least-squares fit on the backbone atoms of that protein to remove global rotational and translational motion.
(TIF)

S11 Fig Backbone RMSD time series for the PPARγ DBD (residues 108–175) and LBD (residues 207–476), and RXRα DBD (residues 132–199) and LBD (residues 230–455) after least-squares fitting to the backbone atoms in the domain. For this analysis, modeled loop regions were excluded.
(TIF)

S1 Table Ligand topologies. (DOCX)
