# Supplementary figures and images for: Phosphorylation of PPARγ Affects the Collective Motions of the PPARγ-RXRα-DNA Complex
Source: PLoS One. 2015 May 8;10(5):e0123984. doi: 10.1371/journal.pone.0123984 (PMC4425662; doi:10.1371/journal.pone.0123984)

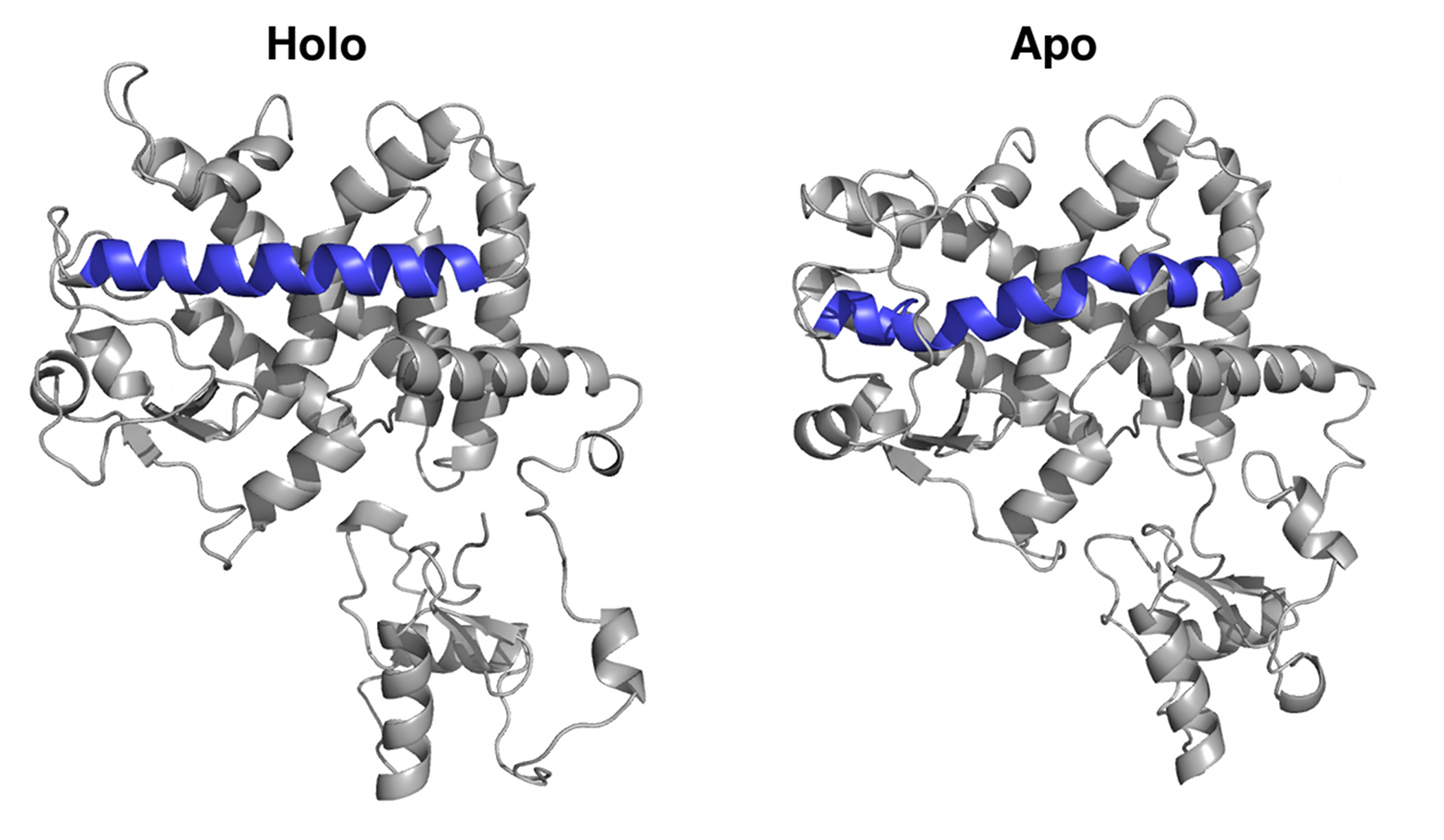

Supplement: S1 Fig — For clarity, only PPARγ is shown in a cartoon representation. H3 is shown in blue to better illustrate its conformation. (TIF) [file pone.0123984.s001.tif]

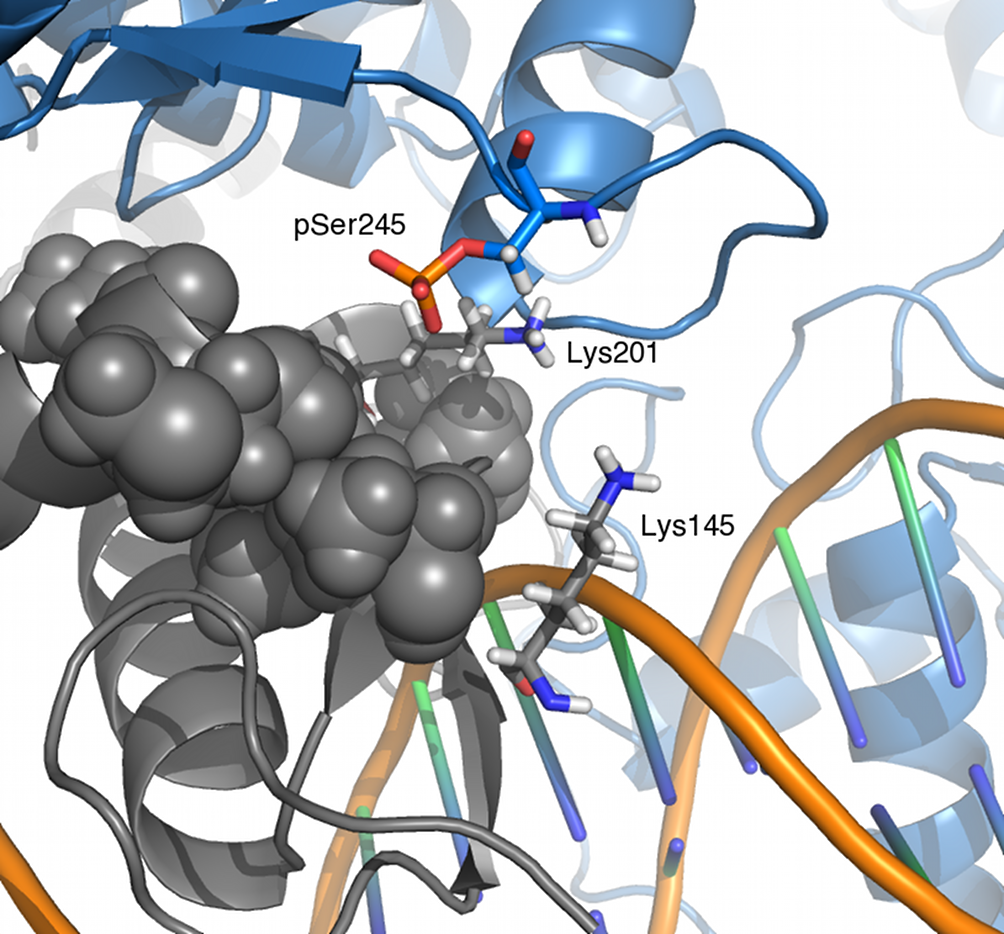

Supplement: S2 Fig — Shown in sticks are pSer245 of PPARγ (blue) as well as Lys145 and Lys201 of RXRα (gray). Hydrophobic residues of RXRα that define the interaction interface between the PPARγ LBD and the RXRα DBD are shown as gray spheres. The double-stranded DNA molecule is shown as a cartoon. (TIF) [file pone.0123984.s002.tif]

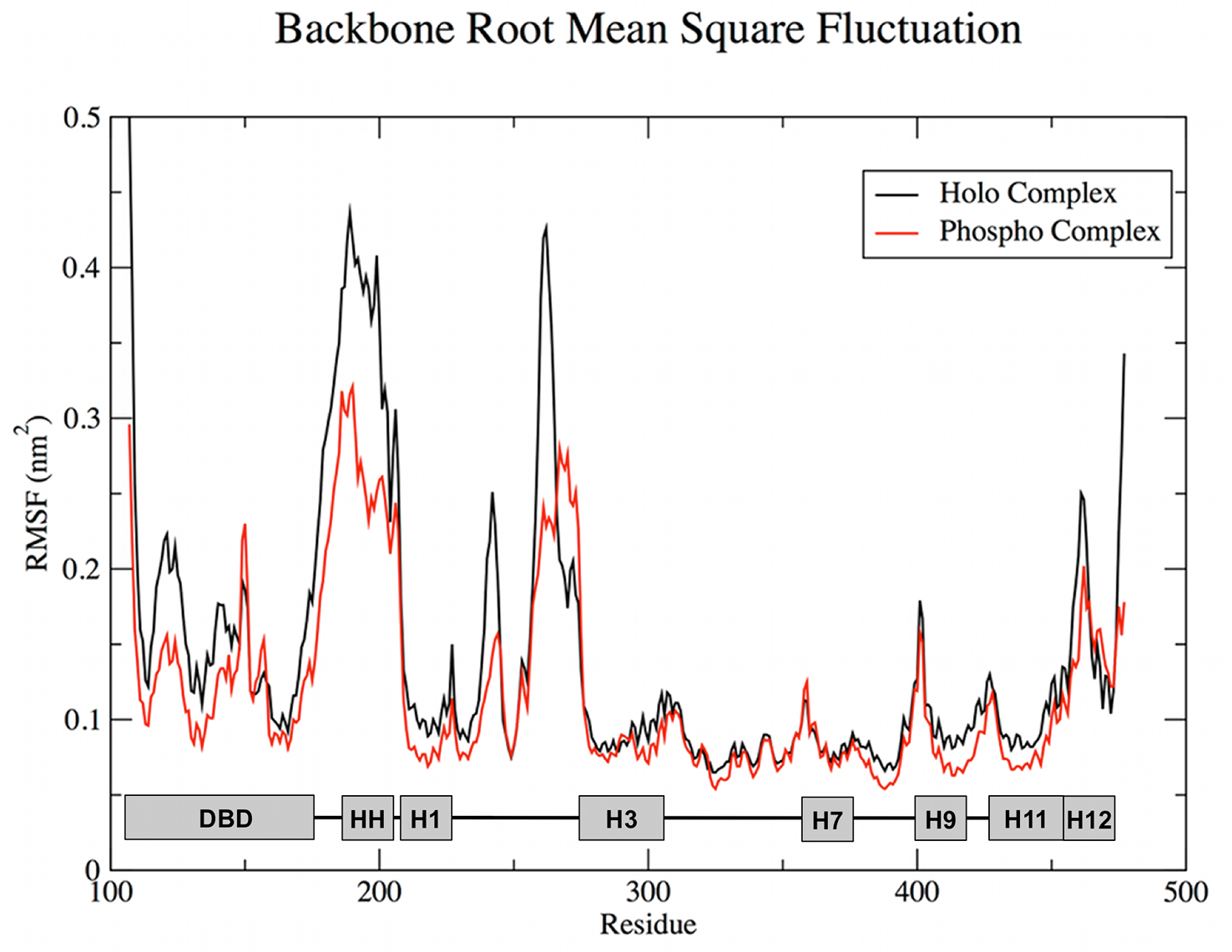

Supplement: S3 Fig — RMSF values for residues flanking pSer245 in the phospho complex are notably reduced relative to Ser245 in the holo complex. The most prominent structural features are labeled. DBD = DNA-binding domain, and helices are denoted as HH (“hinge helix,” see main text), H1, H3, etc. (TIF) [file pone.0123984.s003.tif]

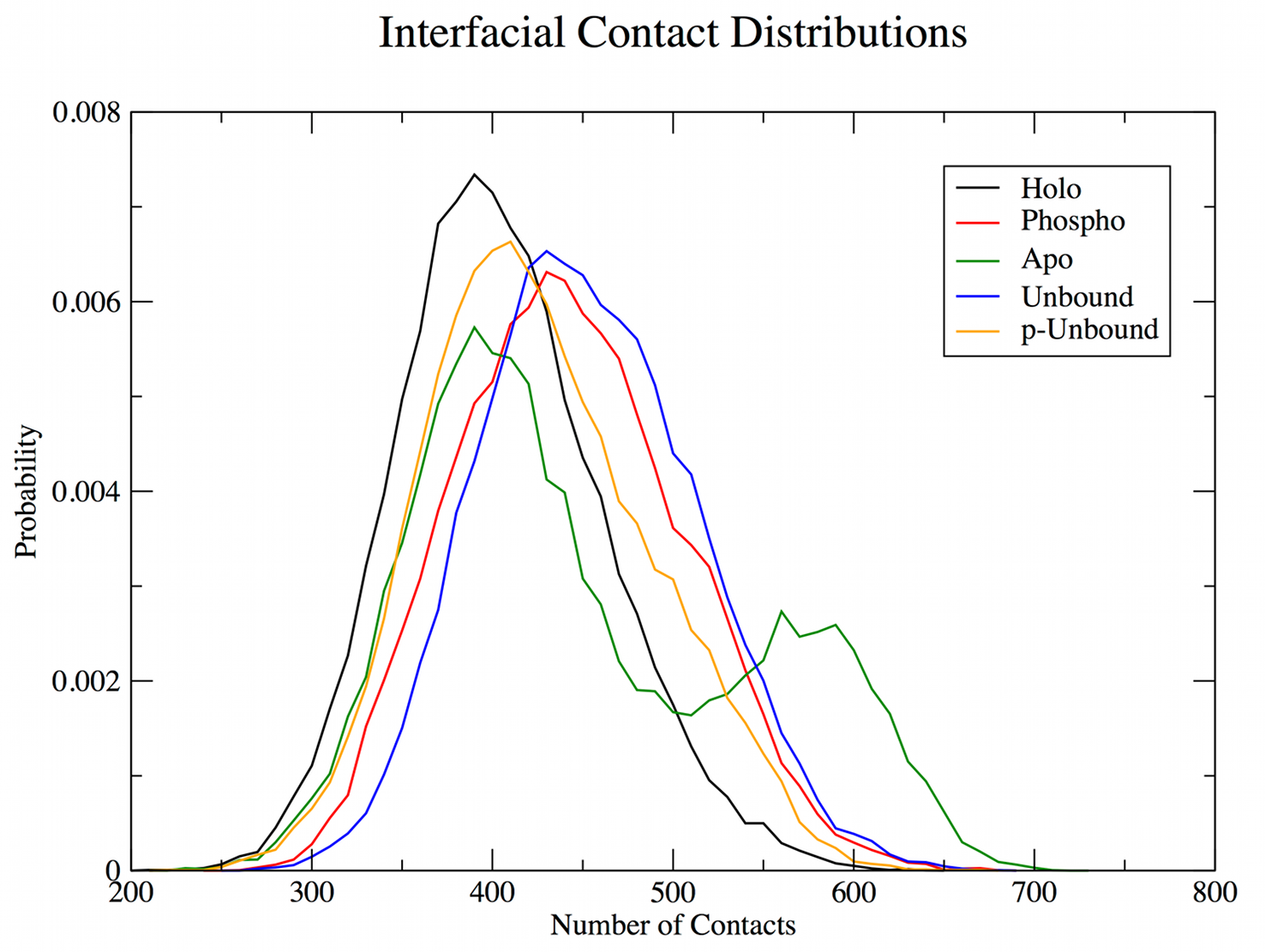

Supplement: S4 Fig — (TIF) [file pone.0123984.s004.tif]

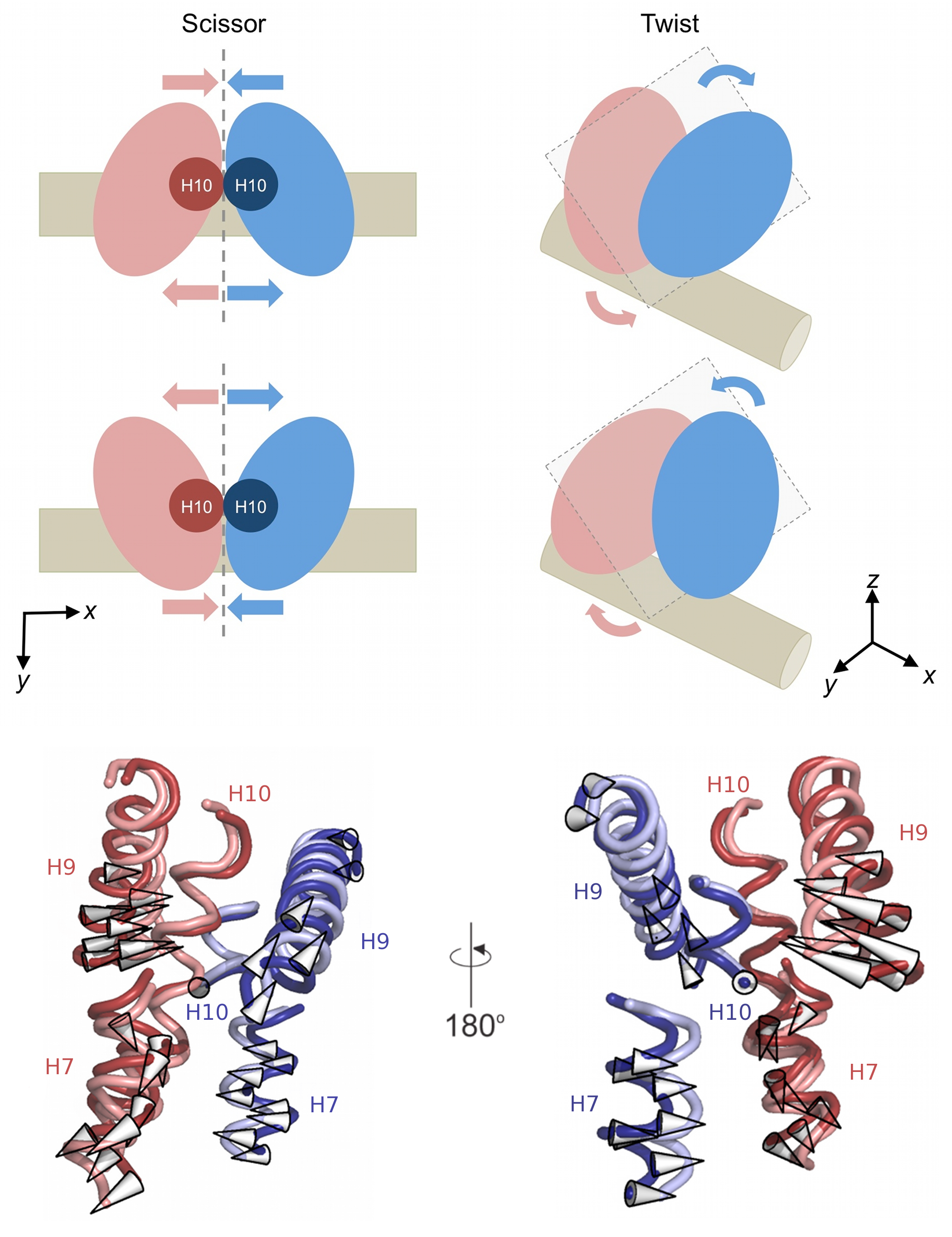

Supplement: S5 Fig — The top panel shows a cartoon of the main scissor and twist motions described in the main text, with PPARγ in blue, RXRα in red, and DNA represented as a tan cylinder. In the bottom panel, PPARγ helices are shown in blue (dark and light representing extremes along the eigenvector), while RXRα helices are shown in red (dark and light again representing extremes). Each helix is labeled, with blue and red indicating PPARγ and RXRα, respectively. The left and right panels are different views, rotated around the vertical axis between the PPARγ and RXRα LBD. H7 of RXRα and H9 of PPARγ “open” while H9 of RXRα and H7 of PPARγ “close,” and vice versa. Arrows indicate the eigenvalues along the eigenvector, with motions only illustrated if they were larger than 0.2 nm. (TIF) [file pone.0123984.s005.tif]

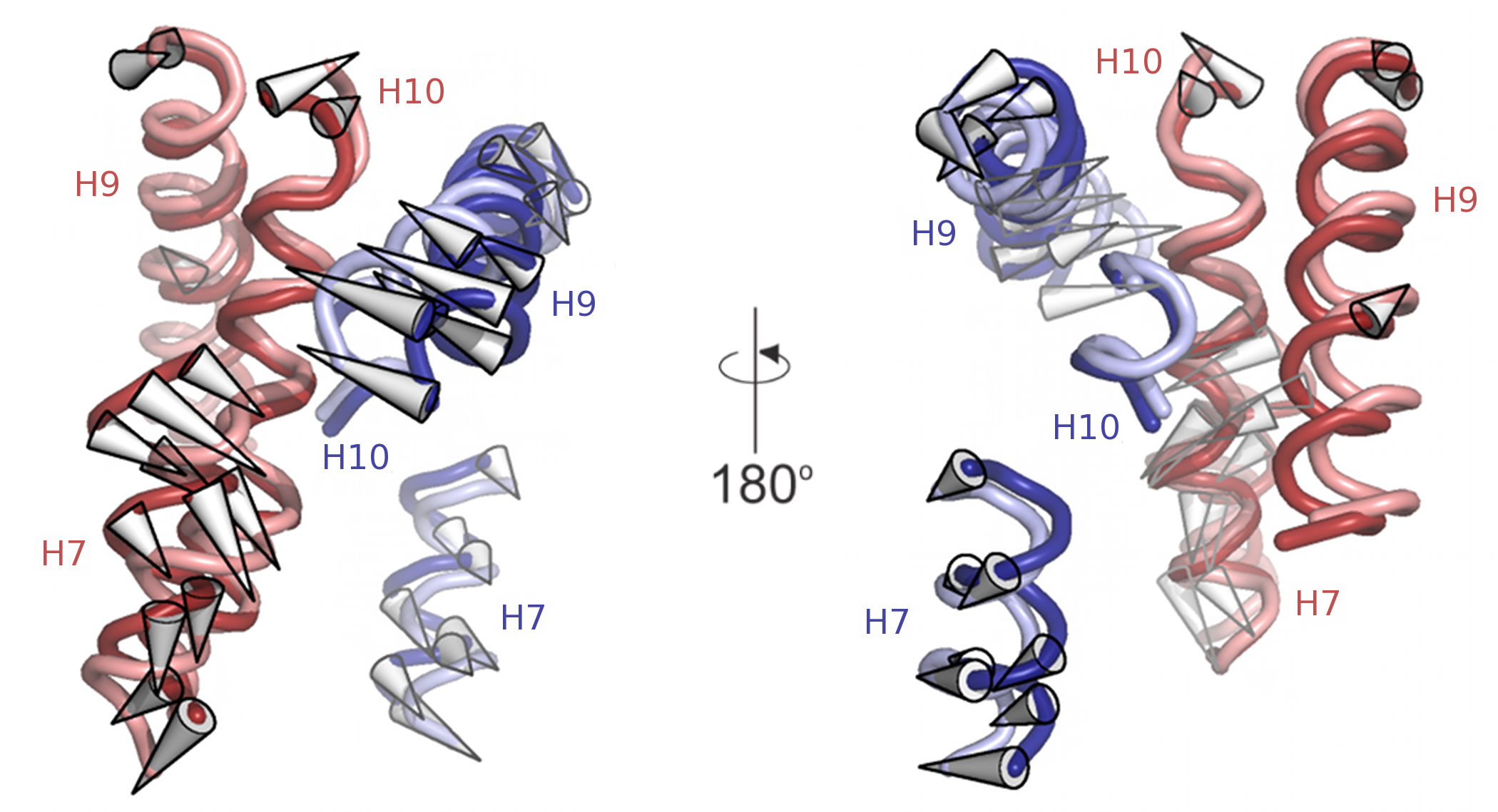

Supplement: S6 Fig — PPARγ helices are shown in blue (dark and light representing extremes along the eigenvector), while RXRα helices are shown in red (dark and light again representing extremes). Each helix is labeled, with blue and red indicating PPARγ and RXRα, respectively. The left and right panels are different views, rotated around the vertical axis between the PPARγ and RXRα LBD. H7 of RXRα and H9 of PPARγ “close” while H9 of RXRα and H7 of PPARγ “open,” though the opening motion is muted compared to the holo complex, and vice versa. Arrows indicate the eigenvalues along the eigenvector, with motions only illustrated if they were larger than 0.2 nm. (TIF) [file pone.0123984.s006.tif]

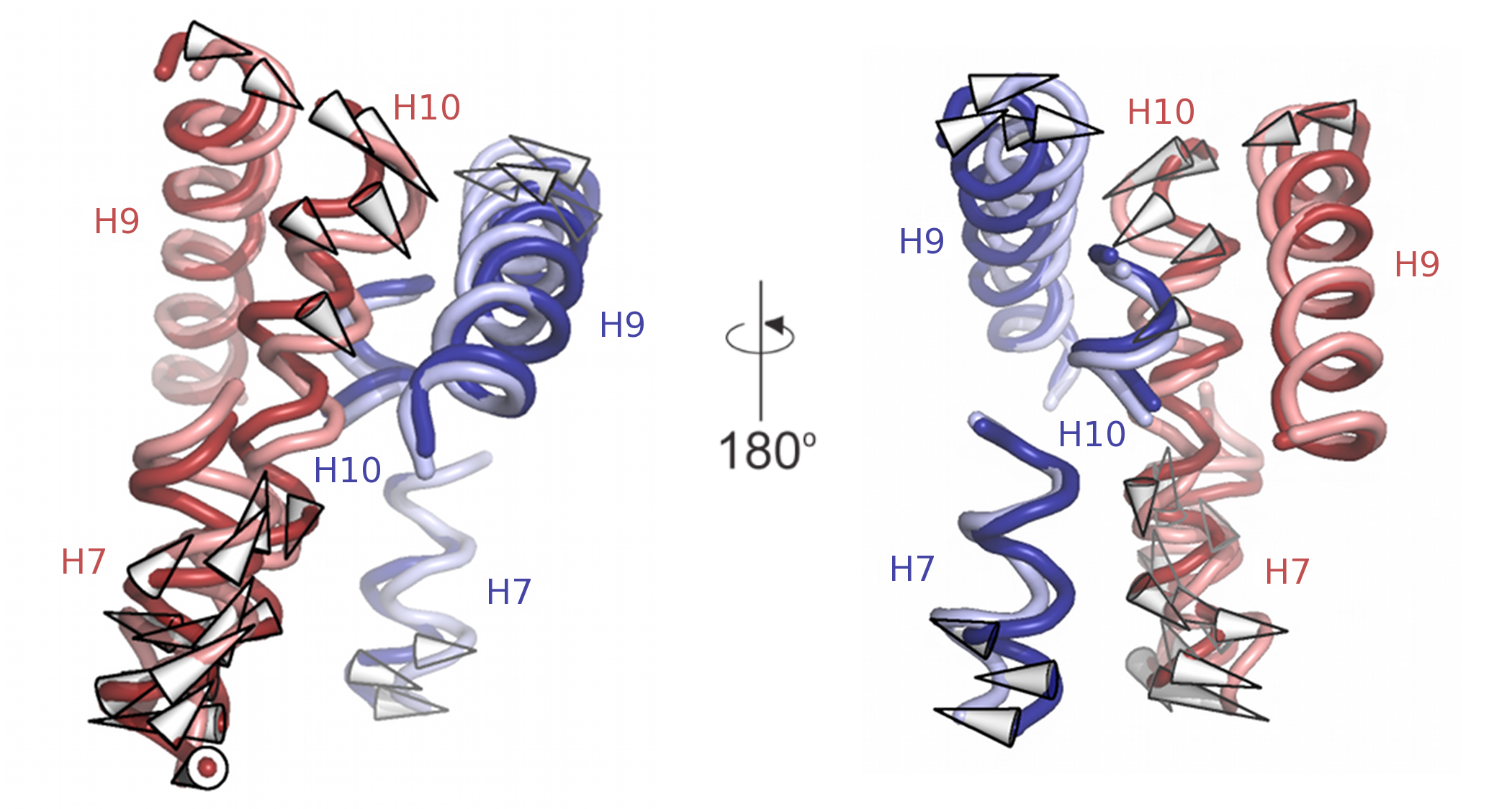

Supplement: S7 Fig — PPARγ helices are shown in blue (dark and light representing extremes along the eigenvector), while RXRα helices are shown in red (dark and light again representing extremes). Each helix is labeled, with blue and red indicating PPARγ and RXRα, respectively. The left and right panels are different views, rotated around the vertical axis between the PPARγ and RXRα LBD. Arrows indicate the eigenvalues along the eigenvector, with motions only illustrated if they were larger than 0.2 nm. (TIF) [file pone.0123984.s007.tif]

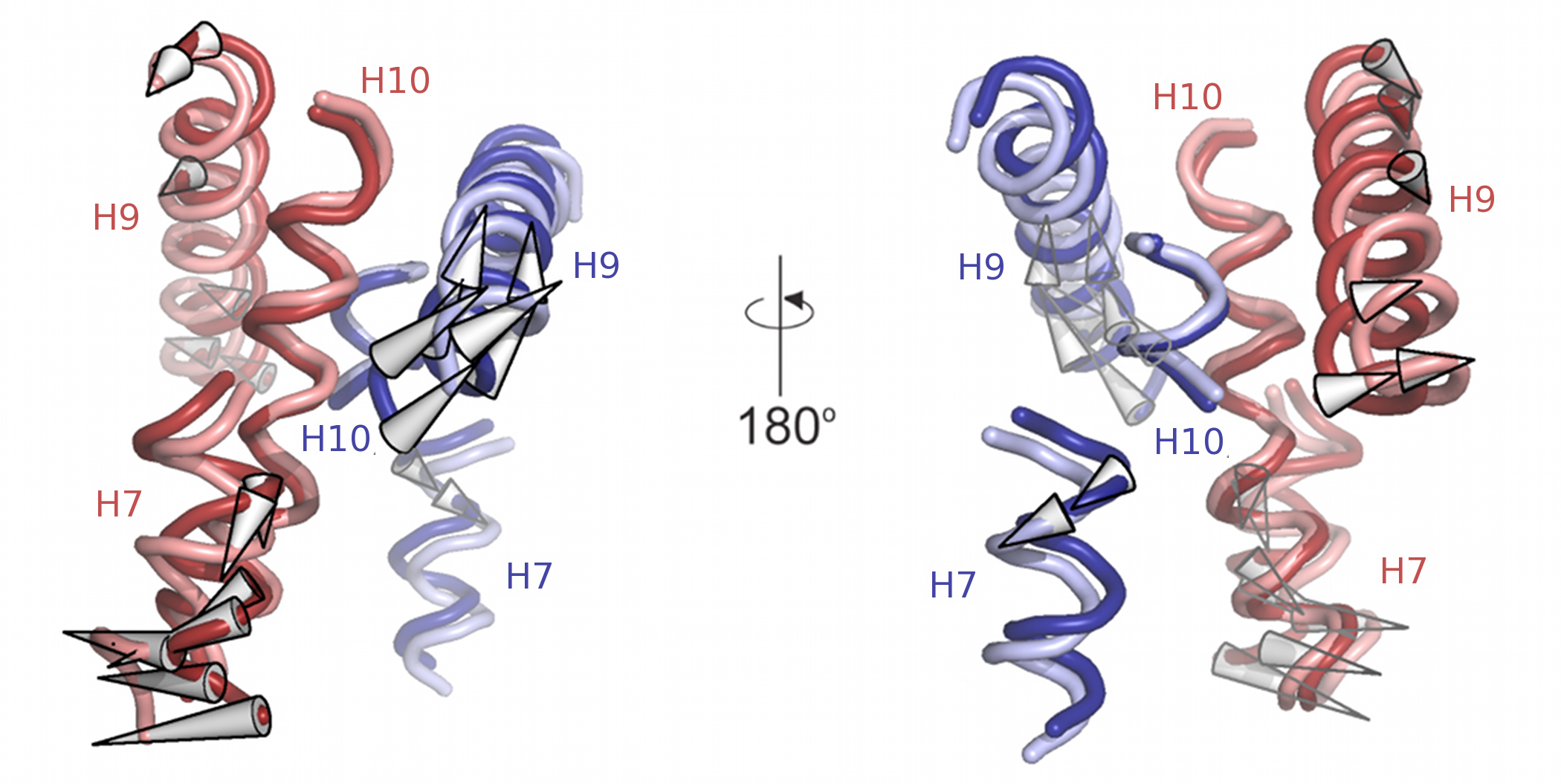

Supplement: S8 Fig — PPARγ helices are shown in blue (dark and light representing extremes along the eigenvector), while RXRα helices are shown in red (dark and light again representing extremes). Each helix is labeled, with blue and red indicating PPARγ and RXRα, respectively. The left and right panels are different views, rotated around the vertical axis between the PPARγ and RXRα LBD. Arrows indicate the eigenvalues along the eigenvector, with motions only illustrated if they were larger than 0.2 nm. (TIF) [file pone.0123984.s008.tif]

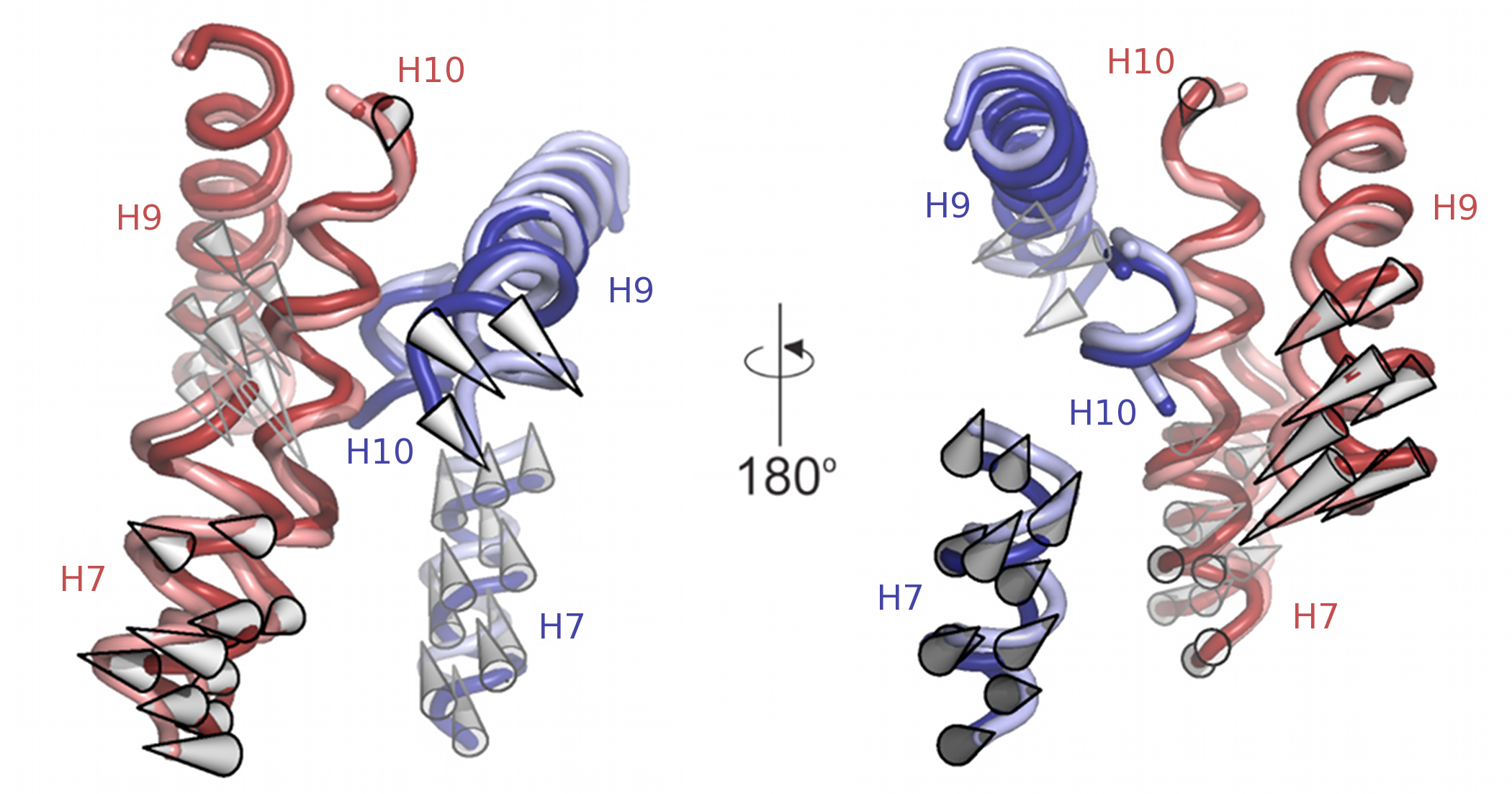

Supplement: S9 Fig — PPARγ helices are shown in blue (dark and light representing extremes along the eigenvector), while RXRα helices are shown in red (dark and light again representing extremes). Each helix is labeled, with blue and red indicating PPARγ and RXRα, respectively. The left and right panels are different views, rotated around the vertical axis between the PPARγ and RXRα LBD. Arrows indicate the eigenvalues along the eigenvector, with motions only illustrated if they were larger than 0.2 nm. (TIF) [file pone.0123984.s009.tif]

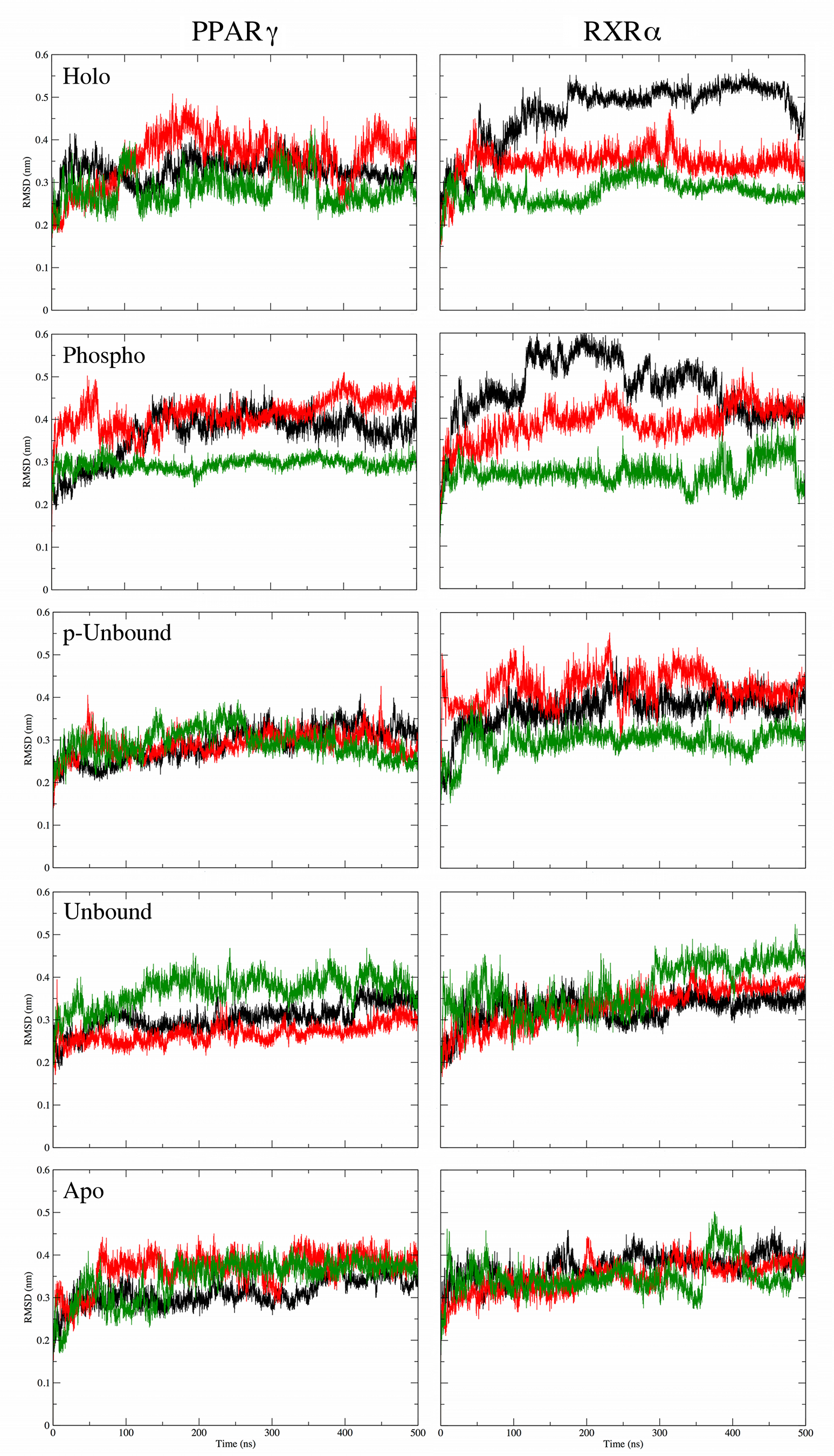

Supplement: S10 Fig — The RMSD for each protein was calculated after performing a least-squares fit on the backbone atoms of that protein to remove global rotational and translational motion. (TIF) [file pone.0123984.s010.tif]

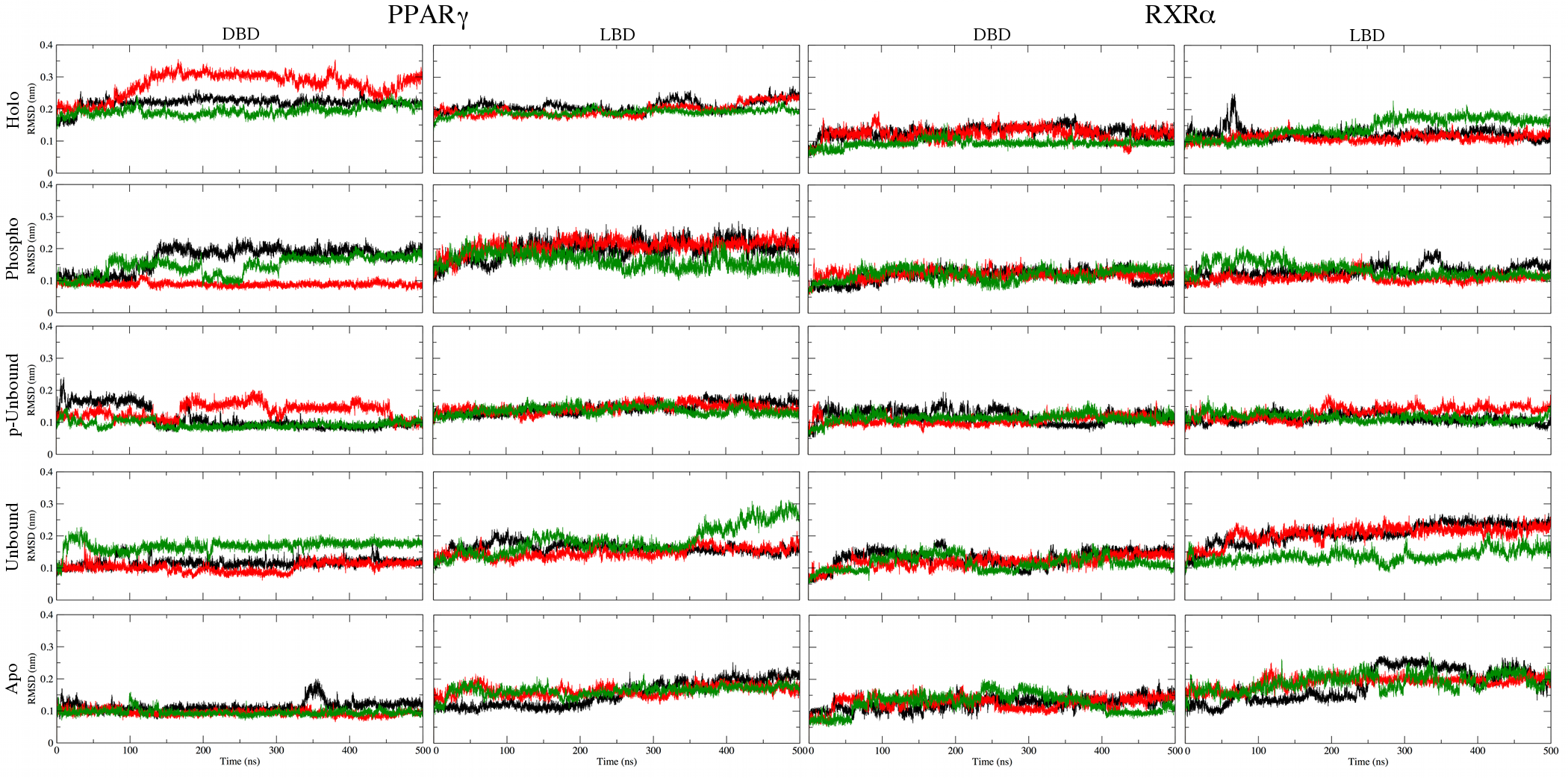

Supplement: S11 Fig — For this analysis, modeled loop regions were excluded. (TIF) [file pone.0123984.s011.tif]
