# Supplementary material for: Phosphorylation of PPARγ Affects the Collective Motions of the PPARγ-RXRα-DNA Complex
Source: PLoS One. 2015 May 8;10(5):e0123984. doi: 10.1371/journal.pone.0123984 (PMC4425662; doi:10.1371/journal.pone.0123984)
Supplement: S1 Table — (DOCX) [file pone.0123984.s012.docx]

; Retinoic acid

[ atomtypes ]

;name bond_type mass charge ptype sigma epsilon

c3 c3 0.0000 0.0000 A 3.39967e-01 4.57730e-01

hc hc 0.0000 0.0000 A 2.64953e-01 6.56888e-02

c c 0.0000 0.0000 A 3.39967e-01 3.59824e-01

ha ha 0.0000 0.0000 A 2.59964e-01 6.27600e-02

cf cf 0.0000 0.0000 A 3.39967e-01 3.59824e-01

c2 c2 0.0000 0.0000 A 3.39967e-01 3.59824e-01

o o 0.0000 0.0000 A 2.95992e-01 8.78640e-01

ce ce 0.0000 0.0000 A 3.39967e-01 3.59824e-01

[ moleculetype ]

; Name nrexcl

REA 3

[ atoms ]

; nr type resnr residue atom cgnr charge mass

1 o 1 REA O1 1 -0.83130 16.000000

2 c 1 REA C15 2 0.89380 12.000000

3 o 1 REA O2 3 -0.82630 16.000000

4 ce 1 REA C14 4 -0.12920 12.000000

5 ha 1 REA H14 5 0.09700 1.000000

6 cf 1 REA C13 6 -0.16020 12.000000

7 c3 1 REA C20 7 -0.04890 12.000000

8 hc 1 REA H20 8 0.00870 1.000000

9 hc 1 REA H8U 9 0.01370 1.000000

10 hc 1 REA H8T 10 0.09270 1.000000

11 cf 1 REA C12 11 -0.04000 12.000000

12 ha 1 REA H12 12 0.10200 1.000000

13 ce 1 REA C11 13 -0.22700 12.000000

14 ha 1 REA H11 14 0.12700 1.000000

15 ce 1 REA C10 15 -0.07900 12.000000

16 ha 1 REA H10 16 0.11000 1.000000

17 cf 1 REA C9 17 -0.14020 12.000000

18 c3 1 REA C19 18 -0.04490 12.000000

19 hc 1 REA H19 19 0.04370 1.000000

20 hc 1 REA H8S 20 0.02870 1.000000

21 hc 1 REA H8R 21 0.02770 1.000000

22 cf 1 REA C8 22 -0.09100 12.000000

23 ha 1 REA H8 23 0.12300 1.000000

24 ce 1 REA C7 24 -0.14600 12.000000

25 ha 1 REA H7 25 0.11500 1.000000

26 ce 1 REA C6 26 -0.07620 12.000000

27 c2 1 REA C5 27 -0.10040 12.000000

28 c3 1 REA C18 28 -0.06590 12.000000

29 hc 1 REA H18 29 0.03870 1.000000

30 hc 1 REA H8Q 30 0.03470 1.000000

31 hc 1 REA H8P 31 0.07270 1.000000

32 c3 1 REA C4 32 -0.04020 12.000000

33 hc 1 REA H42 33 0.04270 1.000000

34 hc 1 REA H41 34 0.04470 1.000000

35 c3 1 REA C3 35 -0.07440 12.000000

36 hc 1 REA H32 36 0.03970 1.000000

37 hc 1 REA H31 37 0.03070 1.000000

38 c3 1 REA C2 38 -0.06840 12.000000

39 hc 1 REA H22 39 0.03170 1.000000

40 hc 1 REA H21 40 0.04170 1.000000

41 c3 1 REA C1 41 -0.00680 12.000000

42 c3 1 REA C16 42 -0.08110 12.000000

43 hc 1 REA H16 43 0.03670 1.000000

44 hc 1 REA H8M 44 0.03570 1.000000

45 hc 1 REA H8L 45 0.02470 1.000000

46 c3 1 REA C17 46 -0.08410 12.000000

47 hc 1 REA H17 47 0.04170 1.000000

48 hc 1 REA H8O 48 0.02170 1.000000

49 hc 1 REA H8N 49 0.04070 1.000000

[ bonds ]

; ai aj funct r k

4 5 1 1.0890e-01 2.8577e+05

7 8 1 1.0920e-01 2.8225e+05

7 9 1 1.0920e-01 2.8225e+05

7 10 1 1.0920e-01 2.8225e+05

11 12 1 1.0890e-01 2.8577e+05

13 14 1 1.0890e-01 2.8577e+05

15 16 1 1.0890e-01 2.8577e+05

18 19 1 1.0920e-01 2.8225e+05

18 20 1 1.0920e-01 2.8225e+05

18 21 1 1.0920e-01 2.8225e+05

22 23 1 1.0890e-01 2.8577e+05

24 25 1 1.0890e-01 2.8577e+05

28 29 1 1.0920e-01 2.8225e+05

28 30 1 1.0920e-01 2.8225e+05

28 31 1 1.0920e-01 2.8225e+05

32 33 1 1.0920e-01 2.8225e+05

32 34 1 1.0920e-01 2.8225e+05

35 36 1 1.0920e-01 2.8225e+05

35 37 1 1.0920e-01 2.8225e+05

38 39 1 1.0920e-01 2.8225e+05

38 40 1 1.0920e-01 2.8225e+05

42 43 1 1.0920e-01 2.8225e+05

42 44 1 1.0920e-01 2.8225e+05

42 45 1 1.0920e-01 2.8225e+05

46 47 1 1.0920e-01 2.8225e+05

46 48 1 1.0920e-01 2.8225e+05

46 49 1 1.0920e-01 2.8225e+05

1 2 1 1.2140e-01 5.4225e+05

2 3 1 1.2140e-01 5.4225e+05

2 4 1 1.4740e-01 3.0443e+05

4 6 1 1.3380e-01 4.7062e+05

6 7 1 1.5050e-01 2.7723e+05

6 11 1 1.4510e-01 3.2677e+05

11 13 1 1.3380e-01 4.7062e+05

13 15 1 1.4510e-01 3.2677e+05

15 17 1 1.3380e-01 4.7062e+05

17 18 1 1.5050e-01 2.7723e+05

17 22 1 1.4510e-01 3.2677e+05

22 24 1 1.3380e-01 4.7062e+05

24 26 1 1.4510e-01 3.2677e+05

26 27 1 1.3390e-01 4.6903e+05

26 41 1 1.5050e-01 2.7723e+05

27 28 1 1.5080e-01 2.7472e+05

27 32 1 1.5080e-01 2.7472e+05

32 35 1 1.5350e-01 2.5363e+05

35 38 1 1.5350e-01 2.5363e+05

38 41 1 1.5350e-01 2.5363e+05

41 42 1 1.5350e-01 2.5363e+05

41 46 1 1.5350e-01 2.5363e+05

[ pairs ]

; ai aj funct

1 5 1

3 5 1

4 8 1

4 9 1

4 10 1

4 12 1

5 7 1

5 11 1

6 14 1

7 12 1

8 11 1

9 11 1

10 11 1

11 16 1

12 14 1

12 15 1

14 16 1

14 17 1

15 19 1

15 20 1

15 21 1

15 23 1

16 18 1

16 22 1

17 25 1

18 23 1

19 22 1

20 22 1

21 22 1

23 25 1

23 26 1

25 27 1

25 41 1

26 29 1

26 30 1

26 31 1

26 33 1

26 34 1

26 39 1

26 40 1

26 43 1

26 44 1

26 45 1

26 47 1

26 48 1

26 49 1

27 36 1

27 37 1

28 33 1

28 34 1

29 32 1

30 32 1

31 32 1

32 39 1

32 40 1

33 36 1

33 37 1

33 38 1

34 36 1

34 37 1

34 38 1

36 39 1

36 40 1

36 41 1

37 39 1

37 40 1

37 41 1

38 43 1

38 44 1

38 45 1

38 47 1

38 48 1

38 49 1

39 42 1

39 46 1

40 42 1

40 46 1

42 47 1

42 48 1

42 49 1

43 46 1

44 46 1

45 46 1

1 6 1

2 7 1

2 11 1

3 6 1

4 13 1

6 15 1

7 13 1

11 17 1

13 18 1

13 22 1

15 24 1

17 26 1

18 24 1

22 27 1

22 41 1

24 28 1

24 32 1

24 38 1

24 42 1

24 46 1

26 35 1

27 38 1

27 42 1

27 46 1

28 41 1

28 35 1

32 41 1

35 42 1

35 46 1

[ angles ]

; ai aj ak funct theta cth

2 4 5 1 1.1726e+02 3.8987e+02

5 4 6 1 1.1829e+02 4.1991e+02

6 7 8 1 1.1098e+02 3.9330e+02

6 7 9 1 1.1098e+02 3.9330e+02

6 7 10 1 1.1098e+02 3.9330e+02

6 11 12 1 1.1590e+02 3.9748e+02

8 7 9 1 1.0835e+02 3.2995e+02

8 7 10 1 1.0835e+02 3.2995e+02

9 7 10 1 1.0835e+02 3.2995e+02

11 13 14 1 1.1829e+02 4.1991e+02

12 11 13 1 1.1798e+02 4.2041e+02

13 15 16 1 1.1590e+02 3.9748e+02

14 13 15 1 1.1590e+02 3.9748e+02

16 15 17 1 1.1829e+02 4.1991e+02

17 18 19 1 1.1098e+02 3.9330e+02

17 18 20 1 1.1098e+02 3.9330e+02

17 18 21 1 1.1098e+02 3.9330e+02

17 22 23 1 1.1590e+02 3.9748e+02

19 18 20 1 1.0835e+02 3.2995e+02

19 18 21 1 1.0835e+02 3.2995e+02

20 18 21 1 1.0835e+02 3.2995e+02

22 24 25 1 1.1829e+02 4.1991e+02

23 22 24 1 1.1798e+02 4.2041e+02

25 24 26 1 1.1590e+02 3.9748e+02

27 28 29 1 1.1049e+02 3.9355e+02

27 28 30 1 1.1049e+02 3.9355e+02

27 28 31 1 1.1049e+02 3.9355e+02

27 32 33 1 1.1049e+02 3.9355e+02

27 32 34 1 1.1049e+02 3.9355e+02

29 28 30 1 1.0835e+02 3.2995e+02

29 28 31 1 1.0835e+02 3.2995e+02

30 28 31 1 1.0835e+02 3.2995e+02

32 35 36 1 1.1005e+02 3.8802e+02

32 35 37 1 1.1005e+02 3.8802e+02

33 32 34 1 1.0835e+02 3.2995e+02

33 32 35 1 1.1005e+02 3.8802e+02

34 32 35 1 1.1005e+02 3.8802e+02

35 38 39 1 1.1005e+02 3.8802e+02

35 38 40 1 1.1005e+02 3.8802e+02

36 35 37 1 1.0835e+02 3.2995e+02

36 35 38 1 1.1005e+02 3.8802e+02

37 35 38 1 1.1005e+02 3.8802e+02

39 38 40 1 1.0835e+02 3.2995e+02

39 38 41 1 1.1005e+02 3.8802e+02

40 38 41 1 1.1005e+02 3.8802e+02

41 42 43 1 1.1005e+02 3.8802e+02

41 42 44 1 1.1005e+02 3.8802e+02

41 42 45 1 1.1005e+02 3.8802e+02

41 46 47 1 1.1005e+02 3.8802e+02

41 46 48 1 1.1005e+02 3.8802e+02

41 46 49 1 1.1005e+02 3.8802e+02

43 42 44 1 1.0835e+02 3.2995e+02

43 42 45 1 1.0835e+02 3.2995e+02

44 42 45 1 1.0835e+02 3.2995e+02

47 46 48 1 1.0835e+02 3.2995e+02

47 46 49 1 1.0835e+02 3.2995e+02

48 46 49 1 1.0835e+02 3.2995e+02

1 2 3 1 1.3038e+02 6.5413e+02

1 2 4 1 1.2292e+02 5.7965e+02

2 4 6 1 1.2641e+02 5.3773e+02

3 2 4 1 1.2292e+02 5.7965e+02

4 6 7 1 1.2203e+02 5.4015e+02

4 6 11 1 1.2433e+02 5.4743e+02

6 11 13 1 1.2433e+02 5.4743e+02

7 6 11 1 1.1801e+02 5.3154e+02

11 13 15 1 1.2405e+02 5.4802e+02

13 15 17 1 1.2405e+02 5.4802e+02

15 17 18 1 1.2203e+02 5.4015e+02

15 17 22 1 1.2433e+02 5.4743e+02

17 22 24 1 1.2433e+02 5.4743e+02

18 17 22 1 1.1801e+02 5.3154e+02

22 24 26 1 1.2405e+02 5.4802e+02

24 26 27 1 1.2308e+02 5.5003e+02

24 26 41 1 1.1659e+02 5.3480e+02

26 27 28 1 1.2302e+02 5.3714e+02

26 27 32 1 1.2302e+02 5.3714e+02

26 41 38 1 1.1122e+02 5.3262e+02

26 41 42 1 1.1122e+02 5.3262e+02

26 41 46 1 1.1122e+02 5.3262e+02

27 26 41 1 1.2289e+02 5.3806e+02

27 32 35 1 1.1144e+02 5.3162e+02

28 27 32 1 1.1652e+02 5.2467e+02

32 35 38 1 1.1063e+02 5.2894e+02

35 38 41 1 1.1063e+02 5.2894e+02

38 41 42 1 1.1063e+02 5.2894e+02

38 41 46 1 1.1063e+02 5.2894e+02

42 41 46 1 1.1063e+02 5.2894e+02

[ dihedrals ]

;i j k l func C0 ... C5

1 2 4 5 3 18.20040 0.00000 -18.20040 0.00000 0.00000 0.00000 ;

3 2 4 5 3 18.20040 0.00000 -18.20040 0.00000 0.00000 0.00000 ;

4 6 7 8 3 0.00000 0.00000 0.00000 0.00000 0.00000 0.00000 ;

4 6 7 9 3 0.00000 0.00000 0.00000 0.00000 0.00000 0.00000 ;

4 6 7 10 3 0.00000 0.00000 0.00000 0.00000 0.00000 0.00000 ;

4 6 11 12 3 8.36800 0.00000 -8.36800 0.00000 0.00000 0.00000 ;

5 4 6 7 3 55.64720 0.00000 -55.64720 0.00000 0.00000 0.00000 ;

5 4 6 11 3 55.64720 0.00000 -55.64720 0.00000 0.00000 0.00000 ;

6 11 13 14 3 55.64720 0.00000 -55.64720 0.00000 0.00000 0.00000 ;

7 6 11 12 3 8.36800 0.00000 -8.36800 0.00000 0.00000 0.00000 ;

8 7 6 11 3 0.00000 0.00000 0.00000 0.00000 0.00000 0.00000 ;

9 7 6 11 3 0.00000 0.00000 0.00000 0.00000 0.00000 0.00000 ;

10 7 6 11 3 0.00000 0.00000 0.00000 0.00000 0.00000 0.00000 ;

11 13 15 16 3 8.36800 0.00000 -8.36800 0.00000 0.00000 0.00000 ;

12 11 13 14 3 55.64720 0.00000 -55.64720 0.00000 0.00000 0.00000 ;

12 11 13 15 3 55.64720 0.00000 -55.64720 0.00000 0.00000 0.00000 ;

14 13 15 16 3 8.36800 0.00000 -8.36800 0.00000 0.00000 0.00000 ;

14 13 15 17 3 8.36800 0.00000 -8.36800 0.00000 0.00000 0.00000 ;

15 17 18 19 3 0.00000 0.00000 0.00000 0.00000 0.00000 0.00000 ;

15 17 18 20 3 0.00000 0.00000 0.00000 0.00000 0.00000 0.00000 ;

15 17 18 21 3 0.00000 0.00000 0.00000 0.00000 0.00000 0.00000 ;

15 17 22 23 3 8.36800 0.00000 -8.36800 0.00000 0.00000 0.00000 ;

16 15 17 18 3 55.64720 0.00000 -55.64720 0.00000 0.00000 0.00000 ;

16 15 17 22 3 55.64720 0.00000 -55.64720 0.00000 0.00000 0.00000 ;

17 22 24 25 3 55.64720 0.00000 -55.64720 0.00000 0.00000 0.00000 ;

18 17 22 23 3 8.36800 0.00000 -8.36800 0.00000 0.00000 0.00000 ;

19 18 17 22 3 0.00000 0.00000 0.00000 0.00000 0.00000 0.00000 ;

20 18 17 22 3 0.00000 0.00000 0.00000 0.00000 0.00000 0.00000 ;

21 18 17 22 3 0.00000 0.00000 0.00000 0.00000 0.00000 0.00000 ;

23 22 24 25 3 55.64720 0.00000 -55.64720 0.00000 0.00000 0.00000 ;

23 22 24 26 3 55.64720 0.00000 -55.64720 0.00000 0.00000 0.00000 ;

25 24 26 27 3 8.36800 0.00000 -8.36800 0.00000 0.00000 0.00000 ;

25 24 26 41 3 8.36800 0.00000 -8.36800 0.00000 0.00000 0.00000 ;

26 27 28 29 3 0.00000 0.00000 0.00000 0.00000 0.00000 0.00000 ;

26 27 28 30 3 0.00000 0.00000 0.00000 0.00000 0.00000 0.00000 ;

26 27 28 31 3 0.00000 0.00000 0.00000 0.00000 0.00000 0.00000 ;

26 27 32 33 3 0.00000 0.00000 0.00000 0.00000 0.00000 0.00000 ;

26 27 32 34 3 0.00000 0.00000 0.00000 0.00000 0.00000 0.00000 ;

26 41 38 39 3 0.65270 1.95811 0.00000 -2.61082 0.00000 0.00000 ;

26 41 38 40 3 0.65270 1.95811 0.00000 -2.61082 0.00000 0.00000 ;

26 41 42 43 3 0.65270 1.95811 0.00000 -2.61082 0.00000 0.00000 ;

26 41 42 44 3 0.65270 1.95811 0.00000 -2.61082 0.00000 0.00000 ;

26 41 42 45 3 0.65270 1.95811 0.00000 -2.61082 0.00000 0.00000 ;

26 41 46 47 3 0.65270 1.95811 0.00000 -2.61082 0.00000 0.00000 ;

26 41 46 48 3 0.65270 1.95811 0.00000 -2.61082 0.00000 0.00000 ;

26 41 46 49 3 0.65270 1.95811 0.00000 -2.61082 0.00000 0.00000 ;

27 32 35 36 3 0.65270 1.95811 0.00000 -2.61082 0.00000 0.00000 ;

27 32 35 37 3 0.65270 1.95811 0.00000 -2.61082 0.00000 0.00000 ;

28 27 32 33 3 0.00000 0.00000 0.00000 0.00000 0.00000 0.00000 ;

28 27 32 34 3 0.00000 0.00000 0.00000 0.00000 0.00000 0.00000 ;

29 28 27 32 3 0.00000 0.00000 0.00000 0.00000 0.00000 0.00000 ;

30 28 27 32 3 0.00000 0.00000 0.00000 0.00000 0.00000 0.00000 ;

31 28 27 32 3 0.00000 0.00000 0.00000 0.00000 0.00000 0.00000 ;

32 35 38 39 3 0.66944 2.00832 0.00000 -2.67776 0.00000 0.00000 ;

32 35 38 40 3 0.66944 2.00832 0.00000 -2.67776 0.00000 0.00000 ;

33 32 35 36 3 0.62760 1.88280 0.00000 -2.51040 0.00000 0.00000 ;

33 32 35 37 3 0.62760 1.88280 0.00000 -2.51040 0.00000 0.00000 ;

33 32 35 38 3 0.66944 2.00832 0.00000 -2.67776 0.00000 0.00000 ;

34 32 35 36 3 0.62760 1.88280 0.00000 -2.51040 0.00000 0.00000 ;

34 32 35 37 3 0.62760 1.88280 0.00000 -2.51040 0.00000 0.00000 ;

34 32 35 38 3 0.66944 2.00832 0.00000 -2.67776 0.00000 0.00000 ;

36 35 38 39 3 0.62760 1.88280 0.00000 -2.51040 0.00000 0.00000 ;

36 35 38 40 3 0.62760 1.88280 0.00000 -2.51040 0.00000 0.00000 ;

36 35 38 41 3 0.66944 2.00832 0.00000 -2.67776 0.00000 0.00000 ;

37 35 38 39 3 0.62760 1.88280 0.00000 -2.51040 0.00000 0.00000 ;

37 35 38 40 3 0.62760 1.88280 0.00000 -2.51040 0.00000 0.00000 ;

37 35 38 41 3 0.66944 2.00832 0.00000 -2.67776 0.00000 0.00000 ;

38 41 42 43 3 0.66944 2.00832 0.00000 -2.67776 0.00000 0.00000 ;

38 41 42 44 3 0.66944 2.00832 0.00000 -2.67776 0.00000 0.00000 ;

38 41 42 45 3 0.66944 2.00832 0.00000 -2.67776 0.00000 0.00000 ;

38 41 46 47 3 0.66944 2.00832 0.00000 -2.67776 0.00000 0.00000 ;

38 41 46 48 3 0.66944 2.00832 0.00000 -2.67776 0.00000 0.00000 ;

38 41 46 49 3 0.66944 2.00832 0.00000 -2.67776 0.00000 0.00000 ;

39 38 41 42 3 0.66944 2.00832 0.00000 -2.67776 0.00000 0.00000 ;

39 38 41 46 3 0.66944 2.00832 0.00000 -2.67776 0.00000 0.00000 ;

40 38 41 42 3 0.66944 2.00832 0.00000 -2.67776 0.00000 0.00000 ;

40 38 41 46 3 0.66944 2.00832 0.00000 -2.67776 0.00000 0.00000 ;

42 41 46 47 3 0.66944 2.00832 0.00000 -2.67776 0.00000 0.00000 ;

42 41 46 48 3 0.66944 2.00832 0.00000 -2.67776 0.00000 0.00000 ;

42 41 46 49 3 0.66944 2.00832 0.00000 -2.67776 0.00000 0.00000 ;

43 42 41 46 3 0.66944 2.00832 0.00000 -2.67776 0.00000 0.00000 ;

44 42 41 46 3 0.66944 2.00832 0.00000 -2.67776 0.00000 0.00000 ;

45 42 41 46 3 0.66944 2.00832 0.00000 -2.67776 0.00000 0.00000 ;

2 6 4 5 3 9.20480 0.00000 -9.20480 0.00000 0.00000 0.00000 ;

13 6 11 12 3 9.20480 0.00000 -9.20480 0.00000 0.00000 0.00000 ;

15 11 13 14 3 9.20480 0.00000 -9.20480 0.00000 0.00000 0.00000 ;

13 17 15 16 3 9.20480 0.00000 -9.20480 0.00000 0.00000 0.00000 ;

24 17 22 23 3 9.20480 0.00000 -9.20480 0.00000 0.00000 0.00000 ;

26 22 24 25 3 9.20480 0.00000 -9.20480 0.00000 0.00000 0.00000 ;

1 2 4 6 3 18.20040 0.00000 -18.20040 0.00000 0.00000 0.00000 ;

2 4 6 7 3 55.64720 0.00000 -55.64720 0.00000 0.00000 0.00000 ;

2 4 6 11 3 55.64720 0.00000 -55.64720 0.00000 0.00000 0.00000 ;

3 2 4 6 3 18.20040 0.00000 -18.20040 0.00000 0.00000 0.00000 ;

4 6 11 13 3 8.36800 0.00000 -8.36800 0.00000 0.00000 0.00000 ;

6 11 13 15 3 55.64720 0.00000 -55.64720 0.00000 0.00000 0.00000 ;

7 6 11 13 3 8.36800 0.00000 -8.36800 0.00000 0.00000 0.00000 ;

11 13 15 17 3 8.36800 0.00000 -8.36800 0.00000 0.00000 0.00000 ;

13 15 17 18 3 55.64720 0.00000 -55.64720 0.00000 0.00000 0.00000 ;

13 15 17 22 3 55.64720 0.00000 -55.64720 0.00000 0.00000 0.00000 ;

15 17 22 24 3 8.36800 0.00000 -8.36800 0.00000 0.00000 0.00000 ;

17 22 24 26 3 55.64720 0.00000 -55.64720 0.00000 0.00000 0.00000 ;

18 17 22 24 3 8.36800 0.00000 -8.36800 0.00000 0.00000 0.00000 ;

22 24 26 27 3 8.36800 0.00000 -8.36800 0.00000 0.00000 0.00000 ;

22 24 26 41 3 8.36800 0.00000 -8.36800 0.00000 0.00000 0.00000 ;

24 26 27 28 3 55.64720 0.00000 -55.64720 0.00000 0.00000 0.00000 ;

24 26 27 32 3 55.64720 0.00000 -55.64720 0.00000 0.00000 0.00000 ;

24 26 41 38 3 0.00000 0.00000 0.00000 0.00000 0.00000 0.00000 ;

24 26 41 42 3 0.00000 0.00000 0.00000 0.00000 0.00000 0.00000 ;

24 26 41 46 3 0.00000 0.00000 0.00000 0.00000 0.00000 0.00000 ;

26 27 32 35 3 0.00000 0.00000 0.00000 0.00000 0.00000 0.00000 ;

26 41 38 35 3 0.65270 1.95811 0.00000 -2.61082 0.00000 0.00000 ;

27 26 41 38 3 0.00000 0.00000 0.00000 0.00000 0.00000 0.00000 ;

27 26 41 42 3 0.00000 0.00000 0.00000 0.00000 0.00000 0.00000 ;

27 26 41 46 3 0.00000 0.00000 0.00000 0.00000 0.00000 0.00000 ;

27 32 35 38 3 0.65270 1.95811 0.00000 -2.61082 0.00000 0.00000 ;

28 27 26 41 3 55.64720 0.00000 -55.64720 0.00000 0.00000 0.00000 ;

28 27 32 35 3 0.00000 0.00000 0.00000 0.00000 0.00000 0.00000 ;

32 27 26 41 3 55.64720 0.00000 -55.64720 0.00000 0.00000 0.00000 ;

32 35 38 41 3 3.68192 3.09616 -2.09200 -3.01248 0.00000 0.00000 ;

35 38 41 42 3 3.68192 3.09616 -2.09200 -3.01248 0.00000 0.00000 ;

35 38 41 46 3 3.68192 3.09616 -2.09200 -3.01248 0.00000 0.00000 ;

4 1 2 3 3 9.20480 0.00000 -9.20480 0.00000 0.00000 0.00000 ;

7 4 6 11 3 9.20480 0.00000 -9.20480 0.00000 0.00000 0.00000 ;

18 15 17 22 3 9.20480 0.00000 -9.20480 0.00000 0.00000 0.00000 ;

27 41 26 24 3 9.20480 0.00000 -9.20480 0.00000 0.00000 0.00000 ;

28 32 27 26 3 9.20480 0.00000 -9.20480 0.00000 0.00000 0.00000 ;

; BVT.13

[ atomtypes ]

;name bond_type mass charge ptype sigma epsilon

n n 0.0000 0.0000 A 3.25000e-01 7.11280e-01

cl cl 0.0000 0.0000 A 3.47094e-01 1.10876e+00

ha ha 0.0000 0.0000 A 2.59964e-01 6.27600e-02

hn hn 0.0000 0.0000 A 1.06908e-01 6.56888e-02

ca ca 0.0000 0.0000 A 3.39967e-01 3.59824e-01

h4 h4 0.0000 0.0000 A 2.51055e-01 6.27600e-02

os os 0.0000 0.0000 A 3.00001e-01 7.11280e-01

nb nb 0.0000 0.0000 A 3.25000e-01 7.11280e-01

c c 0.0000 0.0000 A 3.39967e-01 3.59824e-01

o o 0.0000 0.0000 A 2.95992e-01 8.78640e-01

[ moleculetype ]

; Name nrexcl

PLB 3

[ atoms ]

; nr type resnr residue atom cgnr charge mass

1 nb 1 PLB N27 1 -0.70600 14.000000

2 ca 1 PLB C26 2 0.44420 12.000000

3 h4 1 PLB H26 3 0.01710 1.000000

4 ca 1 PLB C25 4 -0.42760 12.000000

5 ha 1 PLB H25 5 0.14400 1.000000

6 ca 1 PLB C24 6 0.45720 12.000000

7 h4 1 PLB H24 7 0.02410 1.000000

8 nb 1 PLB N23 8 -0.71300 14.000000

9 ca 1 PLB C22 9 0.85830 12.000000

10 os 1 PLB O21 10 -0.33240 16.000000

11 ca 1 PLB C5 11 0.02910 12.000000

12 ca 1 PLB C4 12 -0.11400 12.000000

13 ha 1 PLB H4 13 0.12700 1.000000

14 ca 1 PLB C3 14 -0.17800 12.000000

15 ha 1 PLB H3 15 0.15700 1.000000

16 ca 1 PLB C6 16 -0.07100 12.000000

17 ha 1 PLB H6 17 0.17300 1.000000

18 ca 1 PLB C1 18 -0.15960 12.000000

19 c 1 PLB C7 19 0.91520 12.000000

20 o 1 PLB O8 20 -0.80130 16.000000

21 o 1 PLB O9 21 -0.84430 16.000000

22 ca 1 PLB C2 22 0.08860 12.000000

23 n 1 PLB N10 23 -0.44110 14.000000

24 hn 1 PLB H10 24 0.38950 1.000000

25 c 1 PLB C11 25 0.66870 12.000000

26 o 1 PLB O12 26 -0.62610 16.000000

27 ca 1 PLB C13 27 -0.11360 12.000000

28 ca 1 PLB C14 28 0.05540 12.000000

29 cl 1 PLB Cl19 29 -0.06040 35.500000

30 ca 1 PLB C15 30 -0.13100 12.000000

31 ha 1 PLB H15 31 0.15700 1.000000

32 ca 1 PLB C16 32 0.03140 12.000000

33 cl 1 PLB Cl20 33 -0.10840 35.500000

34 ca 1 PLB C17 34 -0.13600 12.000000

35 ha 1 PLB H17 35 0.14500 1.000000

36 ca 1 PLB C18 36 -0.07400 12.000000

37 ha 1 PLB H18 37 0.15600 1.000000

[ bonds ]

; ai aj funct r k

2 3 1 1.0880e-01 2.8694e+05

4 5 1 1.0870e-01 2.8811e+05

6 7 1 1.0880e-01 2.8694e+05

12 13 1 1.0870e-01 2.8811e+05

14 15 1 1.0870e-01 2.8811e+05

16 17 1 1.0870e-01 2.8811e+05

23 24 1 1.0090e-01 3.4326e+05

30 31 1 1.0870e-01 2.8811e+05

34 35 1 1.0870e-01 2.8811e+05

36 37 1 1.0870e-01 2.8811e+05

1 2 1 1.3420e-01 4.0426e+05

1 9 1 1.3420e-01 4.0426e+05

2 4 1 1.3870e-01 4.0033e+05

4 6 1 1.3870e-01 4.0033e+05

6 8 1 1.3420e-01 4.0426e+05

8 9 1 1.3420e-01 4.0426e+05

9 10 1 1.3730e-01 3.1162e+05

10 11 1 1.3730e-01 3.1162e+05

11 12 1 1.3870e-01 4.0033e+05

11 16 1 1.3870e-01 4.0033e+05

12 14 1 1.3870e-01 4.0033e+05

14 22 1 1.3870e-01 4.0033e+05

16 18 1 1.3870e-01 4.0033e+05

18 19 1 1.4870e-01 2.9263e+05

18 22 1 1.3870e-01 4.0033e+05

19 20 1 1.2140e-01 5.4225e+05

19 21 1 1.2140e-01 5.4225e+05

22 23 1 1.4220e-01 3.1154e+05

23 25 1 1.3450e-01 4.0016e+05

25 26 1 1.2140e-01 5.4225e+05

25 27 1 1.4870e-01 2.9263e+05

27 28 1 1.3870e-01 4.0033e+05

27 36 1 1.3870e-01 4.0033e+05

28 29 1 1.7290e-01 2.7012e+05

28 30 1 1.3870e-01 4.0033e+05

30 32 1 1.3870e-01 4.0033e+05

32 33 1 1.7290e-01 2.7012e+05

32 34 1 1.3870e-01 4.0033e+05

34 36 1 1.3870e-01 4.0033e+05

[ pairs ]

; ai aj funct

1 5 1

2 7 1

9 3 1

3 5 1

3 6 1

5 7 1

5 8 1

7 9 1

10 13 1

10 17 1

11 15 1

12 17 1

13 16 1

13 15 1

13 22 1

14 24 1

15 18 1

15 23 1

17 19 1

17 22 1

18 24 1

24 26 1

24 27 1

25 37 1

27 31 1

27 35 1

28 37 1

29 31 1

30 35 1

31 33 1

31 34 1

32 37 1

33 35 1

35 37 1

1 6 1

1 11 1

2 8 1

2 10 1

9 4 1

6 10 1

8 11 1

9 12 1

9 16 1

10 14 1

10 18 1

11 22 1

11 19 1

12 18 1

12 23 1

14 16 1

14 19 1

14 25 1

16 20 1

16 21 1

16 23 1

18 25 1

19 23 1

20 22 1

21 22 1

22 26 1

22 27 1

23 28 1

23 36 1

25 29 1

25 30 1

25 34 1

26 28 1

26 36 1

27 32 1

28 34 1

28 33 1

29 36 1

29 32 1

30 36 1

33 36 1

[ angles ]

; ai aj ak funct theta cth

1 2 3 1 1.1594e+02 4.3363e+02

2 4 5 1 1.2001e+02 4.0551e+02

3 2 4 1 1.2109e+02 4.0367e+02

4 6 7 1 1.2109e+02 4.0367e+02

5 4 6 1 1.2001e+02 4.0551e+02

7 6 8 1 1.1594e+02 4.3363e+02

11 12 13 1 1.2001e+02 4.0551e+02

11 16 17 1 1.2001e+02 4.0551e+02

12 14 15 1 1.2001e+02 4.0551e+02

13 12 14 1 1.2001e+02 4.0551e+02

15 14 22 1 1.2001e+02 4.0551e+02

17 16 18 1 1.2001e+02 4.0551e+02

22 23 24 1 1.1594e+02 3.9631e+02

24 23 25 1 1.1846e+02 4.1179e+02

27 36 37 1 1.2001e+02 4.0551e+02

28 30 31 1 1.2001e+02 4.0551e+02

31 30 32 1 1.2001e+02 4.0551e+02

32 34 35 1 1.2001e+02 4.0551e+02

34 36 37 1 1.2001e+02 4.0551e+02

35 34 36 1 1.2001e+02 4.0551e+02

1 2 4 1 1.2263e+02 5.7873e+02

1 9 8 1 1.2719e+02 5.9229e+02

1 9 10 1 1.1966e+02 6.0701e+02

2 1 9 1 1.1586e+02 5.7396e+02

2 4 6 1 1.1997e+02 5.6216e+02

4 6 8 1 1.2263e+02 5.7873e+02

6 8 9 1 1.1586e+02 5.7396e+02

8 9 10 1 1.1966e+02 6.0701e+02

9 10 11 1 1.1995e+02 5.2978e+02

10 11 12 1 1.1920e+02 5.8400e+02

10 11 16 1 1.1920e+02 5.8400e+02

11 12 14 1 1.1997e+02 5.6216e+02

11 16 18 1 1.1997e+02 5.6216e+02

12 11 16 1 1.1997e+02 5.6216e+02

12 14 22 1 1.1997e+02 5.6216e+02

14 22 18 1 1.1997e+02 5.6216e+02

14 22 23 1 1.1989e+02 5.6877e+02

16 18 19 1 1.2014e+02 5.4091e+02

16 18 22 1 1.1997e+02 5.6216e+02

18 19 20 1 1.2344e+02 5.7463e+02

18 19 21 1 1.2344e+02 5.7463e+02

18 22 23 1 1.1989e+02 5.6877e+02

19 18 22 1 1.2014e+02 5.4091e+02

20 19 21 1 1.3038e+02 6.5413e+02

22 23 25 1 1.2371e+02 5.3798e+02

23 25 26 1 1.2203e+02 6.3455e+02

23 25 27 1 1.1514e+02 5.7296e+02

25 27 28 1 1.2014e+02 5.4091e+02

25 27 36 1 1.2014e+02 5.4091e+02

26 25 27 1 1.2344e+02 5.7463e+02

27 28 29 1 1.1940e+02 5.2651e+02

27 28 30 1 1.1997e+02 5.6216e+02

27 36 34 1 1.1997e+02 5.6216e+02

28 27 36 1 1.1997e+02 5.6216e+02

28 30 32 1 1.1997e+02 5.6216e+02

29 28 30 1 1.1940e+02 5.2651e+02

30 32 33 1 1.1940e+02 5.2651e+02

30 32 34 1 1.1997e+02 5.6216e+02

32 34 36 1 1.1997e+02 5.6216e+02

33 32 34 1 1.1940e+02 5.2651e+02

[ dihedrals ]

;i j k l func C0 ... C5

1 2 4 5 3 30.33400 0.00000 -30.33400 0.00000 0.00000 0.00000 ;

2 4 6 7 3 30.33400 0.00000 -30.33400 0.00000 0.00000 0.00000 ;

9 1 2 3 3 40.16640 0.00000 -40.16640 0.00000 0.00000 0.00000 ;

3 2 4 5 3 30.33400 0.00000 -30.33400 0.00000 0.00000 0.00000 ;

3 2 4 6 3 30.33400 0.00000 -30.33400 0.00000 0.00000 0.00000 ;

5 4 6 7 3 30.33400 0.00000 -30.33400 0.00000 0.00000 0.00000 ;

5 4 6 8 3 30.33400 0.00000 -30.33400 0.00000 0.00000 0.00000 ;

7 6 8 9 3 40.16640 0.00000 -40.16640 0.00000 0.00000 0.00000 ;

10 11 12 13 3 30.33400 0.00000 -30.33400 0.00000 0.00000 0.00000 ;

10 11 16 17 3 30.33400 0.00000 -30.33400 0.00000 0.00000 0.00000 ;

11 12 14 15 3 30.33400 0.00000 -30.33400 0.00000 0.00000 0.00000 ;

12 11 16 17 3 30.33400 0.00000 -30.33400 0.00000 0.00000 0.00000 ;

13 12 11 16 3 30.33400 0.00000 -30.33400 0.00000 0.00000 0.00000 ;

13 12 14 15 3 30.33400 0.00000 -30.33400 0.00000 0.00000 0.00000 ;

13 12 14 22 3 30.33400 0.00000 -30.33400 0.00000 0.00000 0.00000 ;

14 22 23 24 3 3.76560 0.00000 -3.76560 0.00000 0.00000 0.00000 ;

15 14 22 18 3 30.33400 0.00000 -30.33400 0.00000 0.00000 0.00000 ;

15 14 22 23 3 30.33400 0.00000 -30.33400 0.00000 0.00000 0.00000 ;

17 16 18 19 3 30.33400 0.00000 -30.33400 0.00000 0.00000 0.00000 ;

17 16 18 22 3 30.33400 0.00000 -30.33400 0.00000 0.00000 0.00000 ;

18 22 23 24 3 3.76560 0.00000 -3.76560 0.00000 0.00000 0.00000 ;

24 23 25 26 3 29.28800 -8.36800 -20.92000 0.00000 0.00000 0.00000 ;

24 23 25 27 3 20.92000 0.00000 -20.92000 0.00000 0.00000 0.00000 ;

25 27 36 37 3 30.33400 0.00000 -30.33400 0.00000 0.00000 0.00000 ;

27 28 30 31 3 30.33400 0.00000 -30.33400 0.00000 0.00000 0.00000 ;

27 36 34 35 3 30.33400 0.00000 -30.33400 0.00000 0.00000 0.00000 ;

28 27 36 37 3 30.33400 0.00000 -30.33400 0.00000 0.00000 0.00000 ;

29 28 30 31 3 30.33400 0.00000 -30.33400 0.00000 0.00000 0.00000 ;

30 32 34 35 3 30.33400 0.00000 -30.33400 0.00000 0.00000 0.00000 ;

31 30 32 33 3 30.33400 0.00000 -30.33400 0.00000 0.00000 0.00000 ;

31 30 32 34 3 30.33400 0.00000 -30.33400 0.00000 0.00000 0.00000 ;

32 34 36 37 3 30.33400 0.00000 -30.33400 0.00000 0.00000 0.00000 ;

33 32 34 35 3 30.33400 0.00000 -30.33400 0.00000 0.00000 0.00000 ;

35 34 36 37 3 30.33400 0.00000 -30.33400 0.00000 0.00000 0.00000 ;

2 6 4 5 3 9.20480 0.00000 -9.20480 0.00000 0.00000 0.00000 ;

11 14 12 13 3 9.20480 0.00000 -9.20480 0.00000 0.00000 0.00000 ;

12 22 14 15 3 9.20480 0.00000 -9.20480 0.00000 0.00000 0.00000 ;

11 18 16 17 3 9.20480 0.00000 -9.20480 0.00000 0.00000 0.00000 ;

22 25 23 24 3 9.20480 0.00000 -9.20480 0.00000 0.00000 0.00000 ;

28 32 30 31 3 9.20480 0.00000 -9.20480 0.00000 0.00000 0.00000 ;

32 36 34 35 3 9.20480 0.00000 -9.20480 0.00000 0.00000 0.00000 ;

27 34 36 37 3 9.20480 0.00000 -9.20480 0.00000 0.00000 0.00000 ;

1 2 4 6 3 30.33400 0.00000 -30.33400 0.00000 0.00000 0.00000 ;

1 9 8 6 3 40.16640 0.00000 -40.16640 0.00000 0.00000 0.00000 ;

1 9 10 11 3 7.53120 0.00000 -7.53120 0.00000 0.00000 0.00000 ;

2 1 9 8 3 40.16640 0.00000 -40.16640 0.00000 0.00000 0.00000 ;

2 1 9 10 3 40.16640 0.00000 -40.16640 0.00000 0.00000 0.00000 ;

2 4 6 8 3 30.33400 0.00000 -30.33400 0.00000 0.00000 0.00000 ;

9 1 2 4 3 40.16640 0.00000 -40.16640 0.00000 0.00000 0.00000 ;

4 6 8 9 3 40.16640 0.00000 -40.16640 0.00000 0.00000 0.00000 ;

6 8 9 10 3 40.16640 0.00000 -40.16640 0.00000 0.00000 0.00000 ;

8 9 10 11 3 7.53120 0.00000 -7.53120 0.00000 0.00000 0.00000 ;

9 10 11 12 3 7.53120 0.00000 -7.53120 0.00000 0.00000 0.00000 ;

9 10 11 16 3 7.53120 0.00000 -7.53120 0.00000 0.00000 0.00000 ;

10 11 12 14 3 30.33400 0.00000 -30.33400 0.00000 0.00000 0.00000 ;

10 11 16 18 3 30.33400 0.00000 -30.33400 0.00000 0.00000 0.00000 ;

11 12 14 22 3 30.33400 0.00000 -30.33400 0.00000 0.00000 0.00000 ;

11 16 18 19 3 30.33400 0.00000 -30.33400 0.00000 0.00000 0.00000 ;

11 16 18 22 3 30.33400 0.00000 -30.33400 0.00000 0.00000 0.00000 ;

12 11 16 18 3 30.33400 0.00000 -30.33400 0.00000 0.00000 0.00000 ;

12 14 22 18 3 30.33400 0.00000 -30.33400 0.00000 0.00000 0.00000 ;

12 14 22 23 3 30.33400 0.00000 -30.33400 0.00000 0.00000 0.00000 ;

14 12 11 16 3 30.33400 0.00000 -30.33400 0.00000 0.00000 0.00000 ;

14 22 18 16 3 30.33400 0.00000 -30.33400 0.00000 0.00000 0.00000 ;

14 22 18 19 3 30.33400 0.00000 -30.33400 0.00000 0.00000 0.00000 ;

14 22 23 25 3 3.76560 0.00000 -3.76560 0.00000 0.00000 0.00000 ;

16 18 19 20 3 30.33400 0.00000 -30.33400 0.00000 0.00000 0.00000 ;

16 18 19 21 3 30.33400 0.00000 -30.33400 0.00000 0.00000 0.00000 ;

16 18 22 23 3 30.33400 0.00000 -30.33400 0.00000 0.00000 0.00000 ;

18 22 23 25 3 3.76560 0.00000 -3.76560 0.00000 0.00000 0.00000 ;

19 18 22 23 3 30.33400 0.00000 -30.33400 0.00000 0.00000 0.00000 ;

20 19 18 22 3 30.33400 0.00000 -30.33400 0.00000 0.00000 0.00000 ;

21 19 18 22 3 30.33400 0.00000 -30.33400 0.00000 0.00000 0.00000 ;

22 23 25 26 3 20.92000 0.00000 -20.92000 0.00000 0.00000 0.00000 ;

22 23 25 27 3 20.92000 0.00000 -20.92000 0.00000 0.00000 0.00000 ;

23 25 27 28 3 30.33400 0.00000 -30.33400 0.00000 0.00000 0.00000 ;

23 25 27 36 3 30.33400 0.00000 -30.33400 0.00000 0.00000 0.00000 ;

25 27 28 29 3 30.33400 0.00000 -30.33400 0.00000 0.00000 0.00000 ;

25 27 28 30 3 30.33400 0.00000 -30.33400 0.00000 0.00000 0.00000 ;

25 27 36 34 3 30.33400 0.00000 -30.33400 0.00000 0.00000 0.00000 ;

26 25 27 28 3 30.33400 0.00000 -30.33400 0.00000 0.00000 0.00000 ;

26 25 27 36 3 30.33400 0.00000 -30.33400 0.00000 0.00000 0.00000 ;

27 28 30 32 3 30.33400 0.00000 -30.33400 0.00000 0.00000 0.00000 ;

27 36 34 32 3 30.33400 0.00000 -30.33400 0.00000 0.00000 0.00000 ;

28 27 36 34 3 30.33400 0.00000 -30.33400 0.00000 0.00000 0.00000 ;

28 30 32 33 3 30.33400 0.00000 -30.33400 0.00000 0.00000 0.00000 ;

28 30 32 34 3 30.33400 0.00000 -30.33400 0.00000 0.00000 0.00000 ;

29 28 27 36 3 30.33400 0.00000 -30.33400 0.00000 0.00000 0.00000 ;

29 28 30 32 3 30.33400 0.00000 -30.33400 0.00000 0.00000 0.00000 ;

30 28 27 36 3 30.33400 0.00000 -30.33400 0.00000 0.00000 0.00000 ;

30 32 34 36 3 30.33400 0.00000 -30.33400 0.00000 0.00000 0.00000 ;

33 32 34 36 3 30.33400 0.00000 -30.33400 0.00000 0.00000 0.00000 ;

18 20 19 21 3 9.20480 0.00000 -9.20480 0.00000 0.00000 0.00000 ;

23 27 25 26 3 87.86400 0.00000 -87.86400 0.00000 0.00000 0.00000 ;

27 30 28 29 3 9.20480 0.00000 -9.20480 0.00000 0.00000 0.00000 ;

30 34 32 33 3 9.20480 0.00000 -9.20480 0.00000 0.00000 0.00000 ;
